# Physiological–Biochemical Characteristics and Omics Landscape of Drought Tolerance in *Chlorella* sp. from the Taklamakan Desert of China

**DOI:** 10.3390/ijms27198439

**Published:** 2026-09-22

**Authors:** Kai Han, Yongshun Zhou, Ruyue Tan, Kaile Fan, Mingliang Ding, Junyan Li, Jianqiao Wu, Jianfeng Gao, Fulong Chen

**Affiliations:** 1 College of Life Sciences, Shihezi University, Shihezi 832000, China; 18353169913@163.com (K.H.); zhouyongshunyy@shzu.edu.cn (Y.Z.); tanruyue00@163.com (R.T.); a109815886@126.com (K.F.); dingmingliang24927@163.com (M.D.); lijunyan24927@163.com (J.L.); 18979307965@163.com (J.W.); 2 Key Laboratory of Oasis Town and Mountain-Basin System Ecology, Xinjiang Production and Construction Corps, Shihezi 832000, China

**Keywords:** desert *Chlorella*, drought stress, metabolomics, transcriptomics, molecular mechanisms

## Abstract

Microalgae endemic to desert ecosystems possess exceptional stress-resistance traits; however, the physiological and molecular drought-adaptation mechanisms of desert-dwelling *Chlorella* remain largely uncharacterized. Herein, we combined physiological–biochemical assays, ultrastructural observation, transcriptomics and metabolomics to dissect drought responses in Taklamakan-desert-origin *Chlorella* sp. under polyethylene glycol 6000 (PEG-6000)-simulated mild (10%), moderate (20%), and severe (30%) drought stress, with mechanistic focus on moderate-drought conditions. Moderate drought repressed algal biomass (−26.32%), chlorophyll *a* (−24.40%), maximum photosystem II quantum yield (*Fv*/*Fm*, −16.55%) and effective quantum yield of photosystem II (ΦPSII, −24.35%). Malondialdehyde (MDA) accumulated (+50.69%), alongside elevated activities of superoxide dismutase (SOD, +156.74%) and catalase (CAT, +11.61%). Compatible solutes, including proline, soluble sugars, total protein and total lipid, increased significantly by 39.00%, 39.36%, 26.35% and 23.06%, respectively (*p* < 0.05). Moderate drought triggered cell swelling, partial cell-wall rupture and prominent enlargement of intracellular starch granules and lipid droplets. Transcriptomic profiling demonstrated up-regulation of transcription–translation and DNA-repair machinery, ATP-binding cassette (ABC) transporters, abscisic acid (ABA)/jasmonic acid (JA) signaling, and biosynthetic pathways for protective metabolites, whereas catabolic pathways and mitogen-activated protein kinase (MAPK) signaling were suppressed. Untargeted metabolomics further validated accumulation of trehalose, stress-protective amino acids, vitamins and functional lipids, while chloroplast-membrane-related metabolites and catabolic intermediates declined. This multi-omics study systematically reveals physiological and molecular regulatory cascades underlying drought tolerance in desert *Chlorella* sp., providing candidate genes and metabolites to support microalgal resource exploitation for arid-zone applications.

## 1. Introduction

Microalgae represent ancient photosynthetic organisms [1,2] with biotechnological relevance for bioenergy, forage, environmental restoration, and high-value nutraceutical and pharmaceutical production [3] owing to fast proliferation, high photosynthetic capacity and facile cultivation [4,5]. Abiotic stresses, including salinity, nutrient limitation and drought, are well-known triggers that boost biosynthesis of commercially valuable metabolites in microalgae; accordingly, stress-adaptive capacity is a critical benchmark for both basic algal biology and industrial strain screening [6,7,8].

The Taklamakan Desert (northwestern China) ranks among the largest mobile sand deserts globally, characterized by extreme diurnal-seasonal thermal fluctuation (>70 °C) [9,10], precipitation (<50 mm year^−1^) and massive evaporative potential (2000–3000 mm year^−1^) [11]. Even under such poly-stress conditions (desiccation, high irradiation, temperature extremes, soil salinization and oligotrophy), diverse microalgal assemblages survive within 1–5 cm subsurface sand layers, primarily in dune–interdune lowlands and sheltered micro-niches [12]. Desert microalgae exhibit extraordinary abiotic resilience, yet the mechanistic basis of drought tolerance remains poorly explored compared with aquatic microalgal taxa. Representative aquatic microalgae, including *Anabaena* sp. [13], *Chlorella vulgaris* [14], and *Chlamydomonas reinhardtii* [15], have been extensively characterized for their stress response mechanisms.

Drought stress is arguably the most pervasive and decisive constraint on microalgal survival [16]. It directly impairs cellular metabolism, while high irradiation, temperature extremes, and soil salinization further exacerbate water loss and osmotic imbalance. Therefore, drought tolerance is not merely one among multiple stress responses but a central, integrative trait that underpins the survival of desert microalgae under poly-stress conditions [17,18]. For *Chlorella* sp. inhabiting the Taklamakan Desert, water availability is highly sporadic: water originates mainly from occasional precipitation and condensation, yet it is rapidly lost via evaporation. Drought tolerance allows these cells to endure prolonged desiccation and rapidly resume metabolic and photosynthetic activity once water becomes transiently available. This capacity, underpinned by coordinated transcriptional and metabolic reprogramming, is essential for colonization, persistence, and reproduction within their native desert habitat [19,20].

*Chlorella* strains isolated from desert biological soil crust constitute promising material for dissecting desiccation-tolerance mechanisms. In this work, we subjected a Taklamakan-origin desert *Chlorella* sp. to gradient PEG-6000-mediated drought treatments. Our objectives were: (i) to quantify physiological–biochemical shifts across mild–moderate–severe drought gradients; (ii) to characterize drought-provoked ultrastructural modifications; and (iii) to unravel transcriptional and metabolic reprogramming under moderate drought stress. This multi-layered dataset aims to uncover core physiological and molecular modules conferring drought resistance, identify functional stress-responsive genes and metabolites, and offer theoretical groundwork for exploiting desert microalgal bioresources within arid biomes.

## 2. Results

### 2.1. Effects of Drought Stress on the Growth Rate of Desert Chlorella sp.

Growth performance was monitored by measuring optical density at 680 nm (OD_680_) and dry cell weight (DCW). Moderate-stress (MS, 20% PEG-6000) and severe-stress (HS, 30% PEG-6000) treatments significantly inhibited proliferation of desert *Chlorella* sp. At day 12, OD_680_ values of MS and HS groups were reduced by 49.43% and 62.88% relative to the non-stressed control (CK, 0% PEG-6000; Figure 1A). At day 15, DCW was decreased by 26.32% (MS) and 52.64% (HS), respectively (Figure 1B).

### 2.2. Effects of Drought Stress on Soluble Sugar, Lipid, and Protein Contents

Drought treatments triggered lipid accumulation and transient elevation of soluble sugar and total protein concentrations (Figure 2). Soluble sugar concentrations in MS and HS were significantly higher than CK on days 5 and 10, reaching maximal increments of 39.36% (MS) and 32.78% (HS), followed by a progressive reduction at later time points (Figure 2A). Total lipid content of LS and MS exceeded CK across most sampling points, excluding day 10. At day 5, lipid levels rose by 16.62% (LS), 23.06% (MS), and 42.90% (HS; Figure 2B). Total protein exhibited temporal dynamics comparable to soluble sugars; the MS group peaked at day 10 with a 26.35% increase versus CK and subsequently declined (Figure 2C).

### 2.3. Effects of Drought Stress on Compatible Solutes and Antioxidant-Enzyme Activities

Drought stress significantly altered concentrations of proline (Pro) and malondialdehyde (MDA), as well as activities of superoxide dismutase (SOD) and catalase (CAT; Figure 3). SOD and CAT activities increased rapidly upon drought exposure. MS and HS reached maximal enzyme activities on day 5: SOD increased by 156.74% (MS) and 180.02% (HS); and CAT increased by 11.61% (MS) and 18.19% (HS), with subsequent gradual decreases. For LS treatment, SOD and CAT maxima occurred at day 15, corresponding to 232.29% and 40.69% elevation relative to CK. Proline accumulation displayed treatment-specific temporal profiles. Proline continuously accumulated in LS, peaking on day 25 (+33.05% vs. CK). MS maintained high proline abundance on day 15 (+36.32%) and day 20 (+39.00%). HS achieved its proline maximum as early as day 5 (+30.12%). MDA concentration scaled with drought severity. MS showed its largest relative MDA increment (+50.69%) at day 10; absolute MDA values peaked at day 25, while relative difference to CK decreased to +32.44%. In HS, MDA was already +72.69% higher than CK on day 5 and reached an absolute maximum at day 25 (+53.36% relative to CK).

### 2.4. Effects of Drought Stress on Chlorophyll Content and Chlorophyll-Fluorescence Parameters

Drought led to concentration-dependent declines in chlorophyll *a* and chlorophyll-fluorescence indices (Figure 4). At day 25, MS exhibited reductions relative to CK for electron-transport rate (ETR, −28.57%), maximum PSII photochemical quantum yield (*Fv*/*Fm*, −16.55%), and effective PSII quantum yield (ΦPSII, −24.35%), alongside an elevation in non-regulated non-photochemical energy-dissipation yield (Y(NO), +60.45%). HS produced more severe alterations: ETR (−34.92%), *Fv*/*Fm* (−25.43%), ΦPSII (−34.41%), and Y(NO) (+75.41%). Chlorophyll *a* decreased rapidly after drought imposition; maximal relative losses were detected on day 15: −24.40% for MS and −37.27% for HS versus CK.

### 2.5. Ultrastructural Alterations Induced by Moderate Drought Stress

Microscopic observations were conducted at day 15 under moderate drought stress (MS). At this stage, *Chlorella* sp. TLD 6B exhibited distinct morphological adaptations while maintaining cellular integrity. It is important to note that these structural features are specific to the MS condition at day 15 and may not be directly extrapolated to other stress intensities or temporal stages.

Moderate-drought-stressed (MS, day 15) cells displayed cell-wall wrinkling, occasional rupture, enlarged cell volume, and prominent intracellular deposition of lipids and starch reserves (Figure 5). Scanning electron microscopy (SEM) showed spherical, smooth-surfaced CK cells with diameter = 2.658 ± 0.291 μm (*n* = 60). MS cells presented surface invaginations and partial structural breakage, with significantly augmented cell diameter (3.260 ± 0.376 μm, *n* = 60; *p* < 0.0001). Transmission electron microscopy (TEM) evidenced enlarged starch granules (S) and lipid droplets (L) within MS cells. Laser-scanning confocal microscopy (LSCM) with BODIPY lipid staining supported TEM observations. Normalized lipid-fluorescence intensity for MS (median = 34.21, interquartile range IQR = 29.35–38.80; *n* = 11) was significantly higher than CK (median = 17.91, IQR = 5.54–26.40; *n* = 11; *p* = 0.0009).

### 2.6. Transcriptomic Profiling Under Moderate Drought Stress

Transcriptomic and metabolomic profiling were conducted at day 15 under moderate drought stress (MS). At this stage, *Chlorella* sp. TLD 6B maintained a dynamic equilibrium, exhibiting robust adaptive physiological responses while avoiding irreversible cellular damage. This optimal window allowed for the capture of active drought-responsive molecular signatures. It is important to note that these signatures are specific to the MS condition at day 15 and may not be directly extrapolated to other stress intensities or temporal stages.

The GC content of the assembled transcriptome exceeded 60%. Principal component analysis (PCA; Figure 6A)—PC1 plus PC2—explained 75.25% total variance, separating CK and MS samples. Sample-to-sample correlation analysis confirmed good biological reproducibility; only one pairwise comparison showed a correlation coefficient below 90% (86.3%, Figure S1). According to KEGG functional annotation, 6473 annotated genes were shared between CK and MS (77.35% of total annotated genes, Figure 6B).

Differentially expressed gene (DEG) KEGG enrichment uncovered 89 enriched pathways for up-regulated DEGs and 88 pathways for down-regulated DEGs, covering metabolism, genetic information processing, environmental information processing, cellular processes and organismal system categories (Figures S2 and S3). The top 30 most significantly enriched pathways were prioritized for interpretation. Up-regulated DEGs clustered in RNA-polymerase- and transcription-factor-related processes, ribosome biogenesis, nucleocytoplasmic transport, DNA-damage-related nucleotide-excision repair and homologous-recombination pathways; biosynthetic routes for aromatic amino acids, isoquinoline/tropane/piperidine/pyridine-type alkaloids, nicotinic acid, unsaturated fatty acids, trehalose and exopolysaccharides; and ABC-transporter modules (Figure 7A). Down-regulated DEGs were enriched in photosynthesis, starch–sucrose catabolism, glycolysis, pyruvate catabolism, amino-acid- and lipid-degradation pathways, and MAPK-signaling cascades (Figure 7B).

Heatmaps visualize representative key gene expression patterns (Figure 8A–D). Genes assigned to multiple pathways were plotted only once, at their first occurrence, to eliminate redundancy.

Quantitative real-time PCR (qRT-PCR) validated transcriptomic trends for selected targets (Figure 9A,B). Pearson correlation analysis of log_2_(MS/CK) fold changes between RNA-seq and qRT-qPCR revealed a strong positive correlation (*r* = 0.866, *p* < 0.0001), confirming the reliability of the transcriptomic data (Figure 9C). Trehalose-6-phosphate synthase transcripts were undetectable in CK, hence omitted from qRT-PCR plotting. Full DEG lists are in Supplementary Tables S1 and S2; target-gene descriptions and primer sequences are listed in Supplementary Table S3.

### 2.7. Metabolomic Profiling Under Moderate Drought Stress

Untargeted metabolomics PCA demonstrated separation between CK and MS groups; PC1 + PC2 accounted for 71.1% total variance (Figure 10A). Inter-sample correlation coefficients > 87% supported dataset robustness (Figure S4). In total, 703 metabolites spanning 11 chemical classes were annotated (Figure 10B).

Differential metabolites fell within nine major categories: carbohydrates, vitamins, nucleotides, amino acids, glycerides, free fatty acids, sphingolipids, phosphatidylcholine and phosphatidylethanolamine (Figures S5 and S6). KEGG enrichment identified 39 up-regulated and 27 down-regulated metabolic pathways (Figures S7 and S8). Top 30 enriched-pathway analysis showed induction of translation, ABC-transporter, tropane-piperidine-pyridine-alkaloid, lysine, aromatic-amino-acid, pantothenate-cofactor and nicotinic-acid biosynthetic routes (Figure 11A). Repressed pathways centered on glycolysis, pyruvate catabolism, oxidative phosphorylation and vitamin–catabolic reactions (Figure 11B).

Targeted sugar- and organic-acid-metabolite profiling achieved clear group separation: PCA cumulative variance reached 98.88% (sugars; Figure S9A) and 94.88% (organic acids; Figure S9C). Biological replicate correlation coefficients were higher than 99% for both subsets (Figure S9B,D).

Metabolite relative abundances were normalized against CK (set to 100%). Strong-magnitude differential metabolites (measurement error < 30%) are summarized in Table 1 (metabolite-abbreviation key in Supplementary Table S4). Trehalose (absent in CK), glucose-1,6-diphosphate, glucose-1-phosphate and 1-(sn-glycerol-3-phosphate)-myo-inositol accumulated; arabinonic acid, xylonic acid and 1,5-anhydro-D-sorbitol declined. Stress-induced lipids comprised saturated short-chain fatty acids, long-chain unsaturated fatty acids, monoglycerides, sphingolipid dihydrosphingosine and lysophosphatidylethanolamine (LPE). Reduced lipid species included 2-hydroxy-R-octadecatrienoic acid, low-unsaturation glycerides, linoleoyl-/linolenoyl-containing membrane glycerides and lysophosphatidylcholine (LPC). Tryptophan, arginine, lysine, asparagine and serine increased; leucine, threonine, valine, acetylspermidine and 6-hydroxydopaquinone decreased. Phosphoenolpyruvate, glycerophosphate, 3-phosphoglycerate and maleic acid were elevated; hydroxyvaleric acid, glutaric acid and isovaleric acid were depleted. Nicotinic acid, isonicotinic acid, biotin and ascorbic acid accumulated. 3′,5′-cyclic-AMP and purine-cycle intermediates were up-regulated, while nicotinamide-adenine-dinucleotide-phosphate (NADPH) was down-regulated.

## 3. Discussion

Drought imposed multi-layer physiological perturbations upon desert-origin *Chlorella* sp., including growth suppression, photosystem impairment, and oxidative-stress accumulation. Moderate drought resulted in a ~25–30% loss of biomass and photosynthetic performance, alongside a >50% elevation in MDA. These responses are consistent with the drought-response signatures documented across photosynthetic eukaryotes [21]. Concurrently, the reprogramming of osmoprotectants and lipid reserves—specifically the accumulation of Pro, soluble sugars, and proteins—aligns with their established roles in drought defense [22], which is consistent with the trend of Pro in *C. vulgaris* and *L. boryana* under PEG-6000 stress [23]. The antioxidant system displayed differential activation: SOD activity underwent strong up-regulation, whereas CAT responded moderately, a hierarchical pattern also observed in lichen-symbiotic algae [24]. Furthermore, compared with aquatic references [25,26], our desert isolate sustains measurable growth under 10% PEG-6000, demonstrating intrinsically superior drought resilience.

Cell-diameter enlargement (~22.65% increase) represented a prominent cellular phenotype under moderate drought, a morphological change also reported in *Dunaliella salina* and *Chlorella vulgaris* under PEG-6000 stress [25,27]. Two complementary mechanisms may contribute: cyclin-dependent-kinase-mediated cell-division retardation prolonging cell-growth phases, and enhanced intracellular osmolyte-driven water retention maintaining cellular turgor under osmotic challenge [28,29]. However, *Trebouxia* sp. TR9 and *Coccomyxa simplex* shrank upon desiccation, possibly reflecting different drought regimes (osmotic stress vs. vapor-phase dehydration) [30]. TEM and LSCM verified pronounced enlargement of starch granules and lipid droplets, matching observations reported in terrestrial green microalgae such as *Parachlorella* sp. and *Haematococcus lacustris* under dehydration stress [31,32]. These carbon-rich reserves presumably serve dual functions as osmotic compatible solutes and energy repositories for post-stress recovery.

Transcriptomic and metabolomic analyses reveal that desert *Chlorella* combats drought stress by coordinating rapid stress signaling with genomic protection. Drought induces the up-regulation of RNA polymerase, ribosome biogenesis, and nucleocytoplasmic transport pathways to drive immediate protein synthesis, while concurrently activating nucleotide excision repair and homologous recombination to maintain DNA integrity. These transcriptional responses are reinforced metabolically by enhancing the biosynthesis and suppressing the degradation of key protective compounds [33].

Transcriptomic–metabolomic integration revealed a metabolic-flux redirection strategy: basal catabolic carbon pathways were dampened while protective-metabolite biosynthesis was reinforced. Photosynthetic light-reaction components, Calvin-cycle modules, starch–sucrose degradation, glycolysis and pyruvate catabolism were transcriptionally repressed. Conversely, trehalose and exopolysaccharide biosynthetic genes were induced. The pentose-phosphate pathway constituted a notable exception among carbon-metabolism routes, supplying NADPH to support lipid biosynthesis. Metabolomics detected trehalose exclusively within drought-stressed samples, indicating that trehalose serves as a core drought-responsive compatible solute for desert *Chlorella* sp. (an important metabolite for drought resistance in microalgae [16]). This regulatory scheme is congruent with findings from desert green algae and *Chlamydomonas reinhardtii* under osmotic stress [34,35].

Nitrogen resource allocation was shifted toward stress-mitigating amino acid pools. Biosynthetic pathways for aspartate-family and aromatic amino acids were transcriptionally enhanced, whereas catabolic routes for arginine, proline, and tyrosine were specifically repressed. Metabolite profiling recorded pronounced accumulation of asparagine, tryptophan, arginine, and serine, alongside >2-fold increases in tyrosine, isoleucine, and phenylalanine; conversely, non-stress-protective derivatives such as threonine decreased. Ser accumulation upon desiccation was reported in the cyanobacterium *Tolypothrix scytonemoides* [36]. This specific pattern of amino acid remodeling constitutes a well-documented adaptive module for abiotic-stress tolerance across microalgae and land-plant systems [37,38,39].

Lipid metabolism occupies a central position in drought adaptation for desert *Chlorella* sp. Drought activated lipid-biosynthesis, -elongation and -transport transcripts and suppressed lipid-catabolism genes. Sphingolipids, short-chain saturated fatty acids, long-chain unsaturated fatty acids, glycerides and lysophosphatidylethanolamine accumulated. These lipid species participate in osmotic homeostasis, membrane-damage mitigation, cell-wall cutin formation and stress-signal transduction [40,41,42]. Similarly, increases in triacylglycerol (TAG, a type of glyceride) and monounsaturated fatty acids were detected in *Phaeodactylum tricornutum* under drought stress [43]. By contrast, key chloroplast-membrane-associated glycerolipid species declined, correlating with the measured repression of photosynthetic capacity. Biosynthetic cascades for ascorbic acid, biotin, nicotinic acid and isoquinoline-, tropane-, piperidine-, and pyridine-type alkaloids were transcriptionally up-regulated, accompanied by elevated corresponding metabolite pools. Vitamins and alkaloids function as signaling co-factors and oxidative-damage repair mediators under abiotic stress [44,45,46]; comparable ascorbic-acid-pathway activation was reported for drought-treated *Dunaliella salina* [25]. While alkaloids, sphingolipids, and lysophospholipids have been detected in drought-stressed plants such as *Sophora alopecuroides* [47], *Trifolium repens* [48], and *Bombax ceiba* [49], their accumulation and functions in drought-exposed microalgae remain poorly characterized.

The ABC-transporter gene superfamily emerged as a central regulatory hub. Multiple subfamily members (ABCB, ABCC, ABCD, and ABCG) were significantly induced, mediating hormone translocation, secondary metabolite export, detoxification, and intracellular osmotic homeostasis, consistent with observations in the basal streptophyte alga *Mesostigma viride* [50]. Our dataset indicated that ABA- and JA-signaling modules participated in drought signal transduction. The endogenous ABA and JA biosynthetic pathways are fully retained in *Chlorella* [51,52]. Although several phytohormone biosynthetic genes exhibited upward trends without meeting differential-expression filtering thresholds, multiple pathway-activating metabolites (ascorbic acid, trehalose, phenylalanine, and eicosatetraenoic acid derivatives) accumulated upon drought exposure, in line with reports for *Chlorella sorokiniana* [51]. Regrettably, our metabolomic platform could not directly quantify endogenous ABA and JA; their exact fluctuations in phytohormone levels remain to be validated in dedicated follow-up assays. Intriguingly, core MAPK-signaling genes were significantly down-regulated under moderate drought. Plausible mechanistic explanations include: (i) induction of negative MAPK regulatory proteins [53]; (ii) negative-feedback crosstalk originating from activated ABA signaling [54]; and (iii) resource reallocation under energy-limited stress, prioritizing defensive responses over growth-associated MAPK-dependent cascades [55].

It should be noted that the structural and transcriptomic analyses presented here were performed on MS-treated cells at day 15. Given the time- and concentration-dependent responses observed in physiological and biochemical parameters, different stress intensities (LS or HS) or sampling time points may activate distinct regulatory programs. Therefore, the molecular and structural signatures reported here should be interpreted within this specific experimental context and may not be directly extrapolated to other stress intensities or temporal stages. Nevertheless, the MS condition at day 15 represents a physiologically relevant window in which cells remain viable while mounting active acclimative responses, providing meaningful insight into the drought tolerance mechanisms of desert *Chlorella* sp.

## 4. Materials and Methods

### 4.1. Algal Material and Drought-Stress Culture Setup

The desert *Chlorella* sp. strain TLD 6B was isolated from biological soil crust collected within the Taklamakan Desert (37°36.723′ N, 80°23.442′ E) and previously characterized in-house [56]. To confirm its taxonomic assignment, the 18S rRNA gene was sequenced in this study, and phylogenetic analysis placed strain TLD 6B within the *Chlorella* clade. It was most closely related to *C. vulgaris* (AB162910.1) and *C. vulgaris* var. (AB488577.1), while clearly separated from *Parachlorella*, *Dictyosphaerium*, and other outgroup taxa, confirming that this strain belongs to the genus *Chlorella* (Figure S10, File S5). Cultivation was performed using Bold’s Basal Medium (BBM) [57] under conditions described previously for similar *Chlorella* species [58]. Drought stress was simulated by adding PEG-6000 to final mass–volume concentrations: CK (0%, control), LS (10%, mild drought stress), MS (20%, moderate drought stress), and HS (30%, severe drought stress). Initial inoculation OD_680_ was adjusted to ~0.2. Each treatment contained three independent biological replicates; cultivation lasted for 25 days. Full medium recipes and culture parameters are documented in Supplementary File S1.

Polyethylene glycol 6000 (PEG-6000) was used to simulate drought stress. As a high-molecular-weight, chemically inert polymer with a hydrodynamic radius of 1.92–2.65 nm [59], PEG-6000 cannot penetrate the algal cell wall or plasma membrane [60]. It lowers medium water potential and reduces water availability for algal cells, mimicking natural osmotic drought, and enables precise, stable, and reproducible water potential control in liquid culture [43]. Although PEG-6000 has been reported to alter cell wall mechanical properties in some plant systems [61], such effects occur under different concentrations or exposure durations relative to those applied in the present study. Under our MS treatment (20% PEG-6000), no cell lysis or loss of membrane integrity was observed, indicating that osmotic stress dominates in this system. The cell wall alterations detected by microscopy are therefore most likely adaptive responses to osmotic stress rather than direct PEG-6000-induced chemical damage.

### 4.2. Physiological–Biochemical Parameter Assays

Cell growth was tracked by OD_680_ spectrophotometry and gravimetric dry-cell-weight (DCW) measurement [62]. Soluble sugar, total lipid, total protein, proline, and MDA were quantified using anthrone–sulfuric acid [63], phosphovanillin [64], Coomassie Brilliant Blue [65], acid ninhydrin [66], and thiobarbituric acid [67] methods, respectively. CAT and SOD activities were assayed via the ultraviolet absorbance decay of hydrogen peroxide [68] and nitro-blue-tetrazolium photoreduction [69], respectively. Chlorophyll-fluorescence parameters were acquired with a handheld fluorometer (AP 110/C, PSI, Brno, Czech Republic) [70], and Chlorophyll-*a* was extracted by ethanol [71]. OD_680_ was logged daily; other biochemical indices were sampled every five days.

### 4.3. Microstructural Observation

Day-15-cultured CK and MS algal samples were prepared for ultrastructural imaging, adapting protocols published for the lichen-associated green alga *Trebouxia lynnae* [72], and images were captured using SEM (SU8010, Hitachi, Tokyo, Japan) and TEM (HT7700, Hitachi, Tokyo, Japan). Neutral lipid staining used BODIPY 500/510 C1, C12 (MedChemExpress, Monmouth Junction, NJ, USA) [73]; LSCM imaging was completed on the Leica Stellaris confocal system (Leica Microsystems, Wetzlar, Germany). Cell diameter and normalized lipid-fluorescence intensity were quantified by ImageJ.JS (version 1.54r, https://ij.js.org/, accessed on 15 July 2026). Complete sample-preparation workflows are given in Supplementary File S2.

### 4.4. Transcriptomic and Metabolomic Analyses

Day 15 CK and MS samples were submitted for multi-omics profiling. Transcriptome sequencing was performed by Majorbio Biomedical (Shanghai, China), following the testing conditions and analytical methods used for common *Chlorella* under cadmium stress [74]. Untargeted metabolomics was outsourced to Metware Biotechnology (Wuhan, China), with reference to the methods described for common *Chlorella* under dehydration stress [75]. Targeted sugar- and organic-acid measurements were conducted by Sanshu Biotechnology (Nantong, China). All experimental handling, aliquoting, labeling, statistical analysis and figure generation were performed independently by the authors. Sequencing parameters, bioinformatic pipelines and detection settings are detailed in Supplementary File S3 (transcriptomics) and Supplementary File S4 (metabolomics). qRT-PCR validation was implemented for selected target transcripts.

### 4.5. Statistical Analysis

All physiological–biochemical data are presented as mean ± standard deviation (SD) of three biological replicates. IBM SPSS Statistics 26.0 (IBM, Armonk, NY, USA) was used for statistical evaluation. One-way analysis of variance (ANOVA) followed by Tukey’s post hoc multiple-comparison test was applied; significance threshold was set at *p* < 0.05. Lipid-droplet normalized-fluorescence-intensity data did not satisfy normality assumptions and are reported as median plus interquartile range (IQR); group comparison used the Mann–Whitney U test. Figures were plotted using OriginPro 2024 (OriginLab Corporation, Northampton, MA, USA).

## 5. Conclusions

In summary, this multi-omics investigation systematically characterizes drought-adaptive physiology and molecular regulatory cascades within Taklamakan-desert-origin *Chlorella* sp. Mild PEG-simulated drought scarcely perturbed algal performance; moderate-to-severe drought repressed growth, diminished photosynthetic capacity, and provoked lipid-peroxidation damage. To counteract stress injury, the alga boosted SOD-CAT antioxidant capacity and accumulated osmoprotective molecules, including proline, soluble sugars, proteins, and lipids. Moderate drought provoked cellular enlargement, partial cell-wall disruption, and pronounced expansion of starch granules and lipid-storage droplets. Multi-omics evidence demonstrates transcriptional activation of transcription–translation machineries and DNA-damage-repair modules. Repression of MAPK signaling, together with activation of ABC-transporter and putative ABA/JA signal-transduction modules, facilitates resource redirection from vegetative growth toward stress defense. Metabolic flux is re-routed away from primary carbon catabolism toward the biosynthesis of protective carbohydrates, amino acids, alkaloids, functional lipids, and vitamins, with corresponding catabolic routes simultaneously attenuated. Collectively, this integrated dataset not only advances the mechanistic comprehension of drought tolerance in desert microalgae but also identifies key drought-responsive genes and protective metabolites, offering a theoretical foundation and valuable genetic resources for the biotechnological exploitation of microalgal bioresources in arid-zone ecosystems. These conclusions are specific to MS-treated cells at day 15, and further studies covering a wider range of stress intensities and sampling time points are required to generalize these findings.

## Figures and Tables

**Figure 1:**
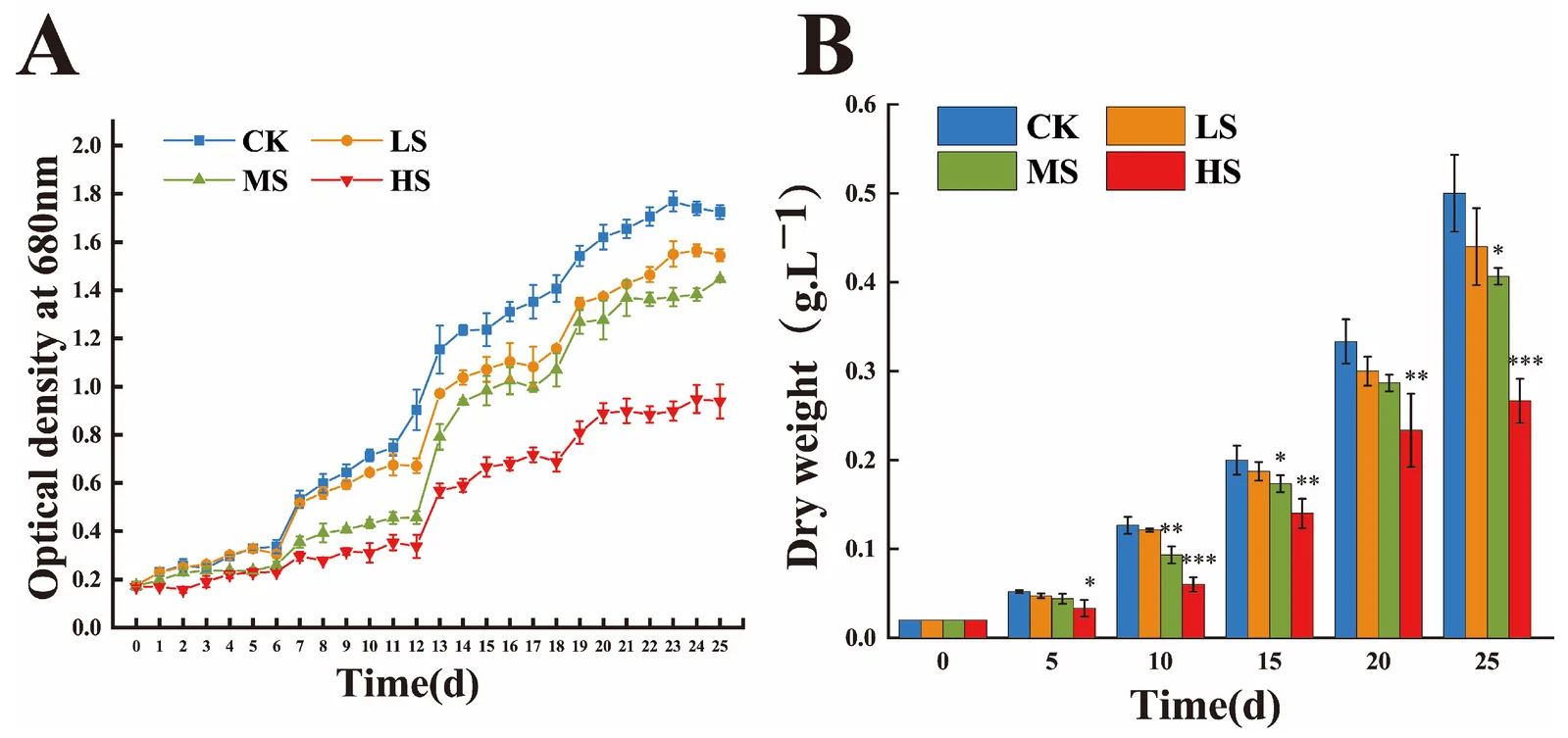
Growth changes of desert *Chlorella* sp. under drought stress. (**A**) Growth curves based on OD_680_ absorbance; (**B**) dry cell weight (DCW). CK: control; LS: mild drought stress; MS: moderate drought stress; HS: severe drought stress. * *p* < 0.05; ** *p* < 0.01; *** *p* < 0.001.

**Figure 2:**
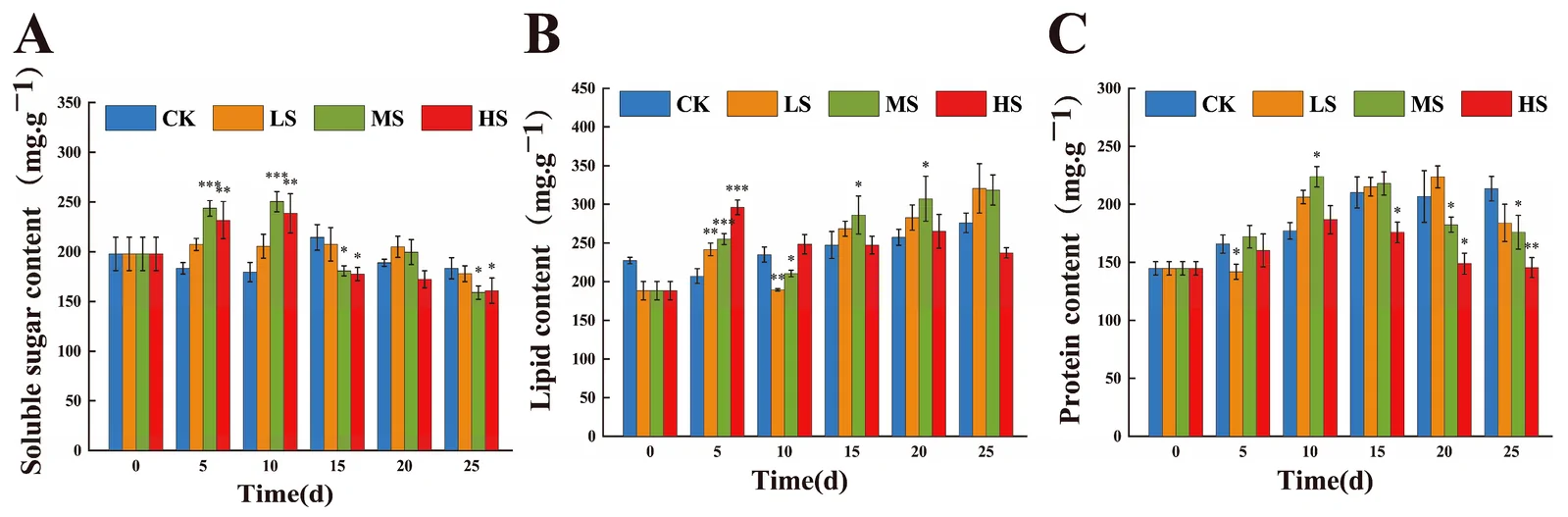
Contents of soluble sugars, total lipids and total protein in desert *Chlorella* sp. under drought stress. (**A**) Soluble sugars; (**B**) total lipids; (**C**) total protein. CK: control; LS: mild drought stress; MS: moderate drought stress; HS: severe drought stress. * *p* < 0.05; ** *p* < 0.01; *** *p* < 0.001.

**Figure 3:**
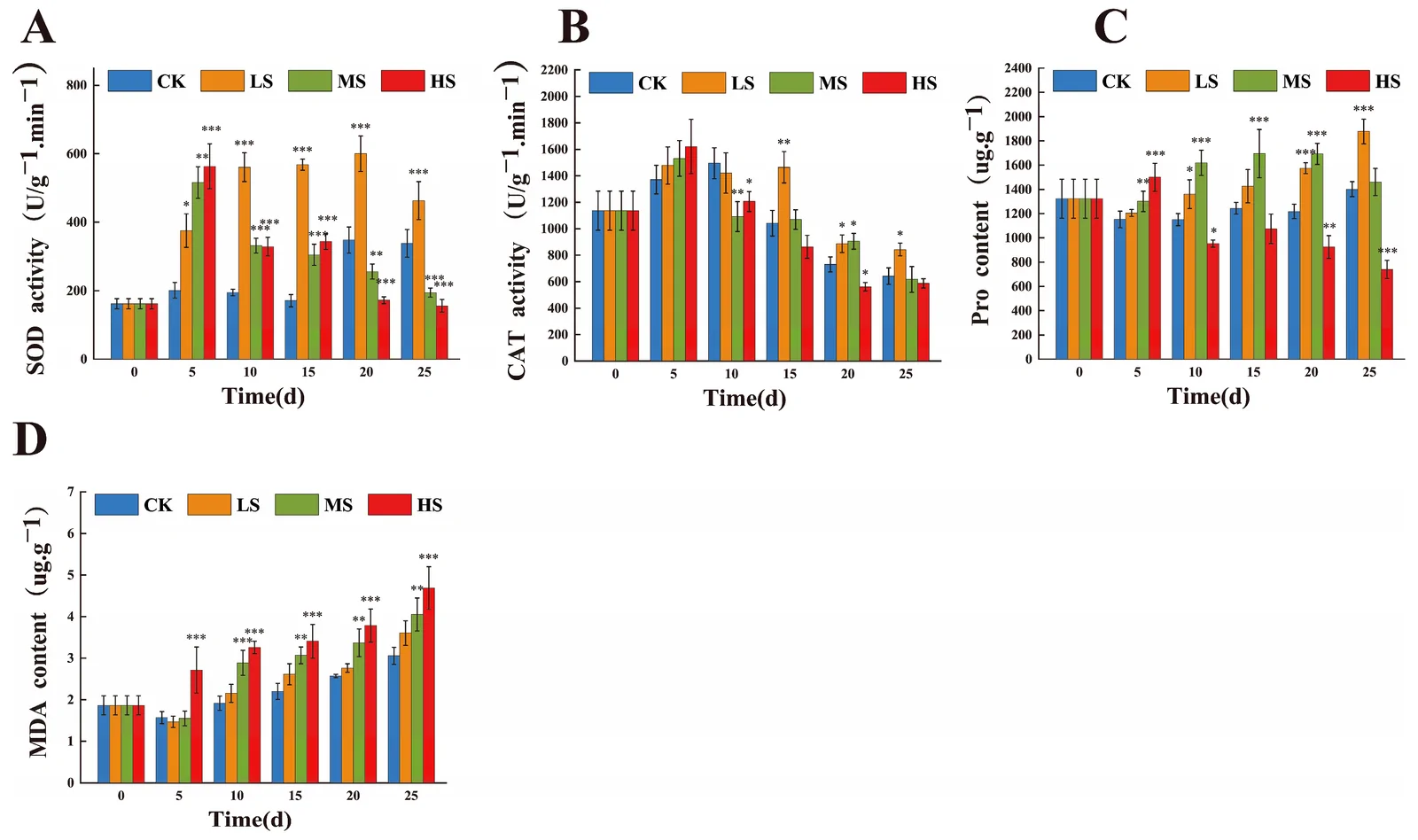
Stress-responsive metabolites and antioxidant-enzyme activities of desert *Chlorella* sp. under drought stress. (**A**) Superoxide dismutase (SOD) activity; (**B**) catalase (CAT) activity; (**C**) proline (Pro) content; (**D**) malondialdehyde (MDA) content. CK: control; LS: mild drought stress; MS: moderate drought stress; HS: severe drought stress. * *p* < 0.05; ** *p* < 0.01; *** *p* < 0.001.

**Figure 4:**
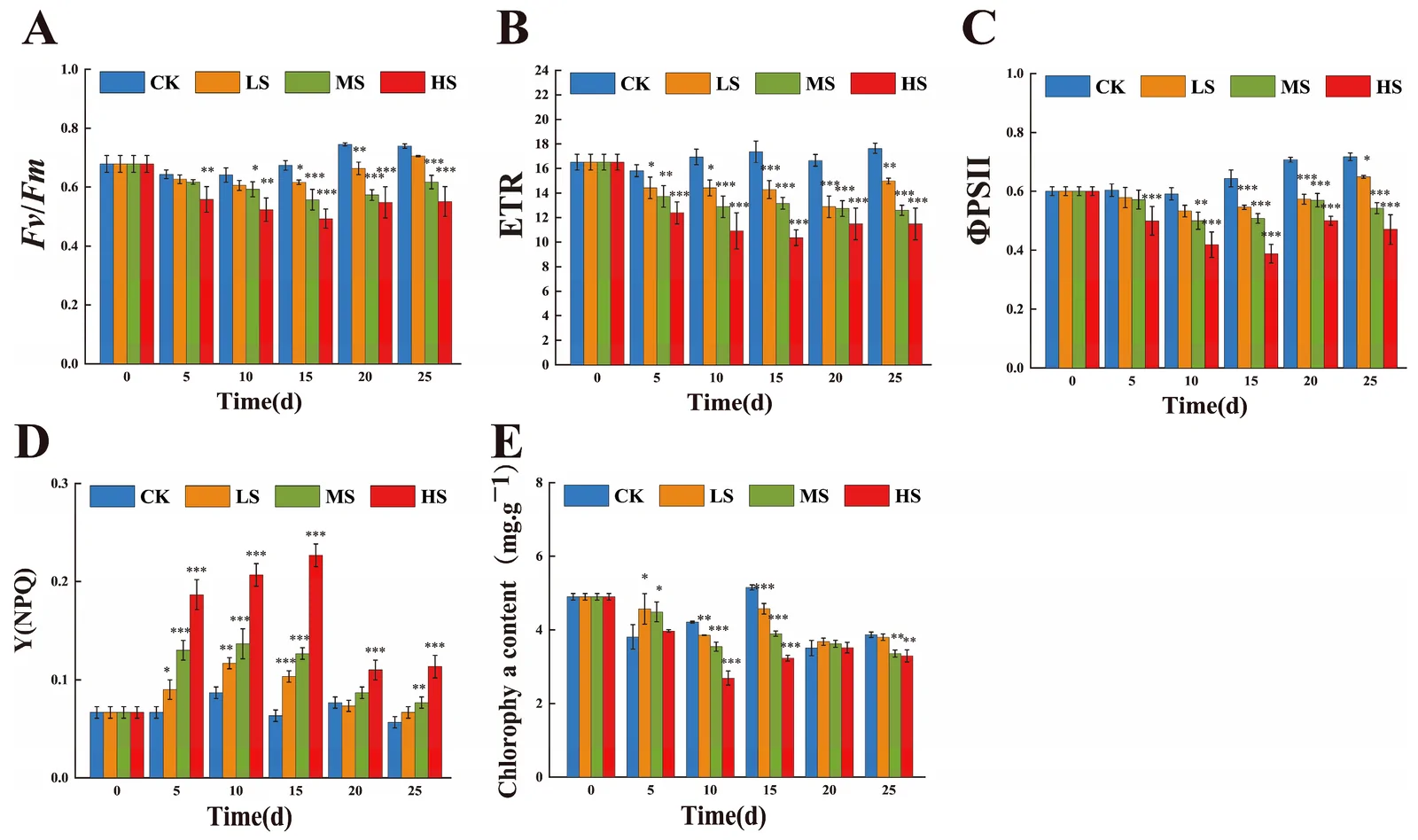
Chlorophyll *a* content and chlorophyll-fluorescence parameters of desert *Chlorella* sp. under drought stress. (**A**) Maximum PSII photochemical quantum yield (*Fv*/*Fm*); (**B**) electron-transport rate (ETR); (**C**) effective PSII quantum yield (ΦPSII); (**D**) non-regulated non-photochemical dissipation yield Y (NO); (**E**) chlorophyll *a* concentration. CK: control; LS: mild drought stress; MS: moderate drought stress; HS: severe drought stress. * *p* < 0.05; ** *p* < 0.01; *** *p* < 0.001.

**Figure 5:**
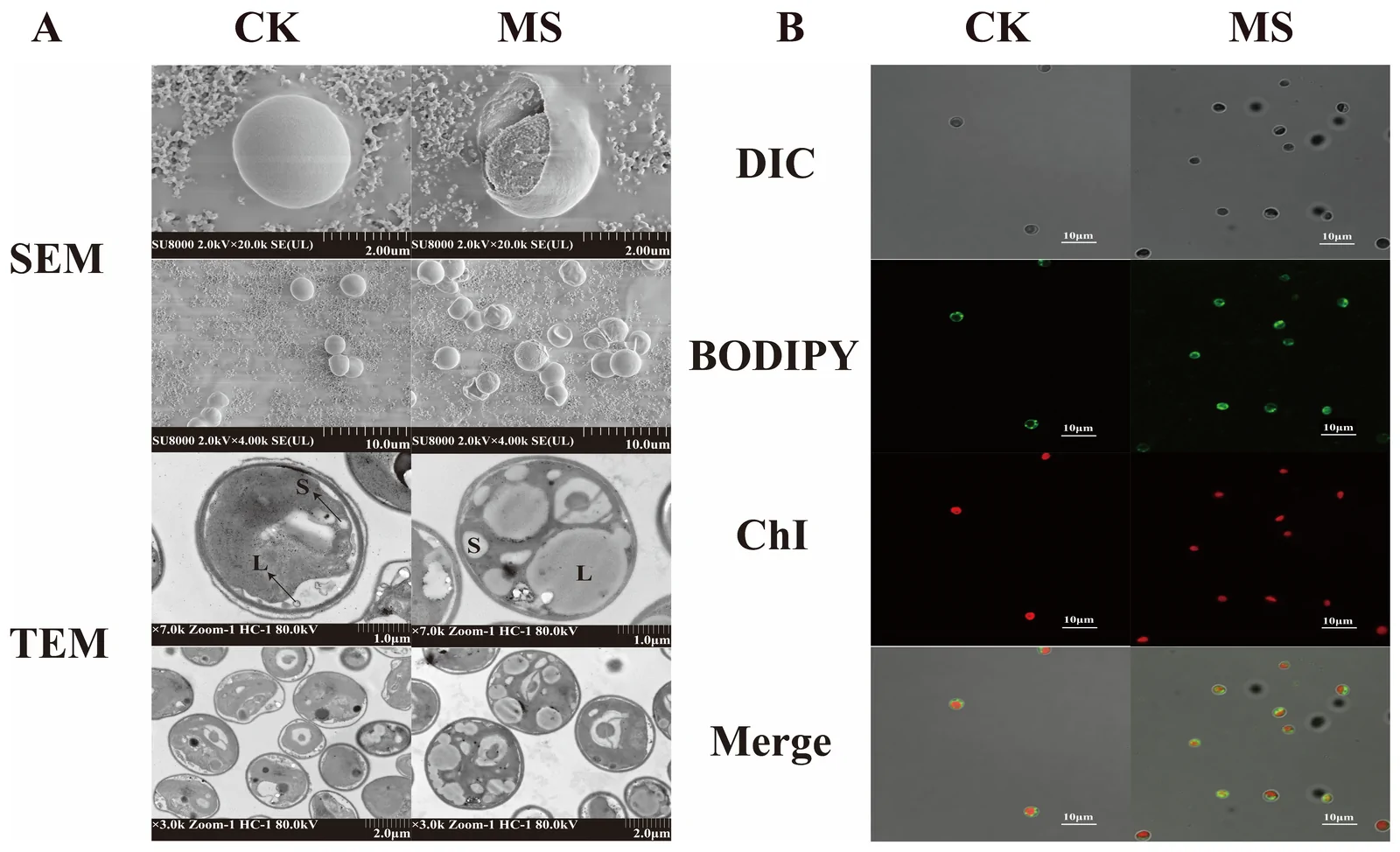
Ultrastructural characterization of desert *Chlorella* sp. under moderate drought stress. (**A**) SEM and TEM micrographs. SEM accelerating voltage = 2.0 kV, magnifications ×20,000 and ×4000; TEM accelerating voltage = 80.0 kV, magnifications ×7000 and ×2500. L: lipid droplet; S: starch granule. (**B**) LSCM multi-channel fluorescence imaging. DIC: differential-interference-contrast bright-field; BODIPY: lipid-targeted fluorescence probe; Chl: chlorophyll autofluorescence; Merge: composite overlay. Objective magnification 100×. CK: control; MS: moderate drought stress.

**Figure 6:**
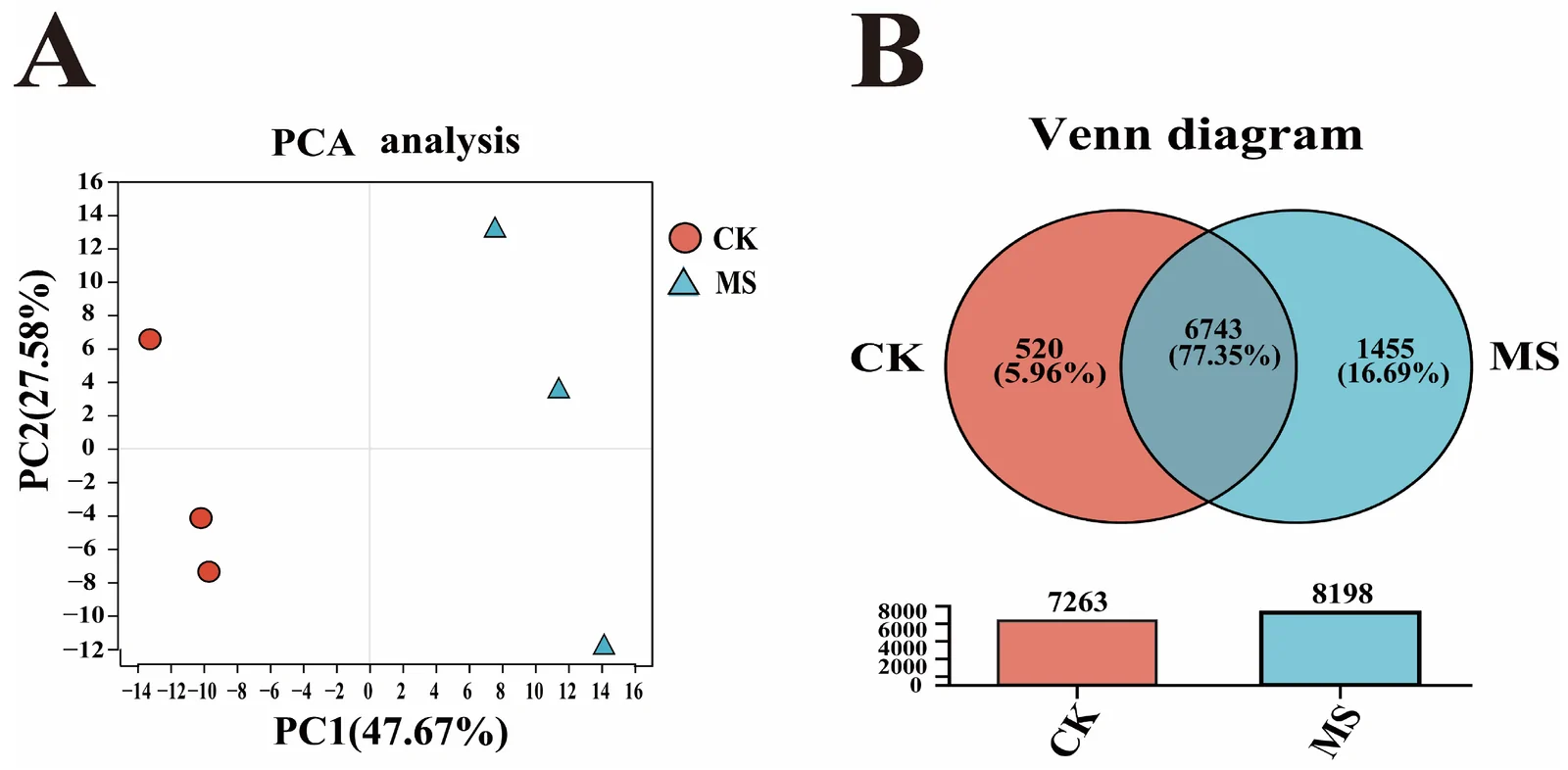
PCA and Venn analysis of transcriptomic changes in desert *Chlorella* sp. under moderate drought stress (MS vs. CK). (**A**) PCA score plot; (**B**) Venn diagram for KEGG-annotated gene sets. CK: control; MS: moderate drought stress.

**Figure 7:**
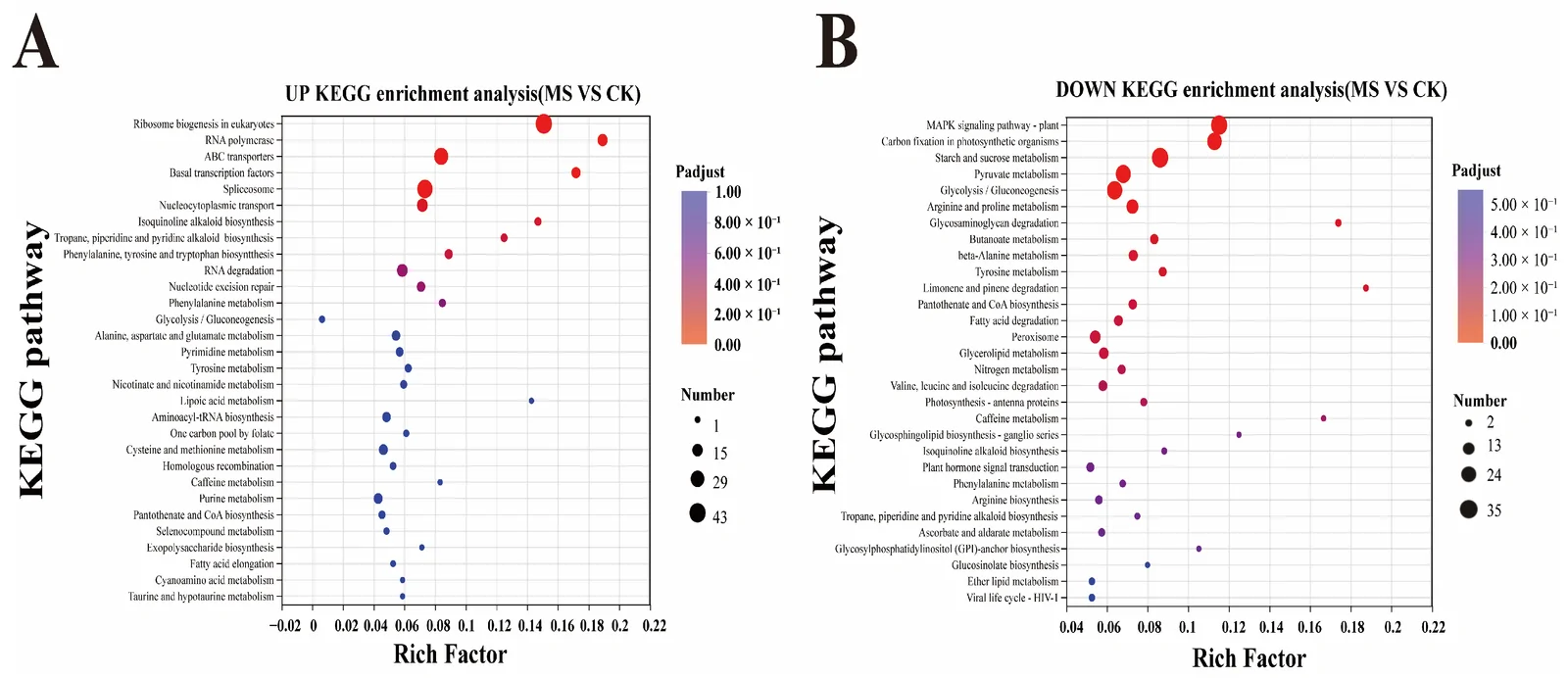
KEGG enrichment analysis of DEGs in desert *Chlorella* sp. under moderate drought stress (MS vs. CK). (**A**) KEGG enrichment scatter plot for up-regulated DEGs; (**B**) KEGG enrichment scatter plot for down-regulated DEGs. CK: control; MS: moderate drought stress.

**Figure 8:**
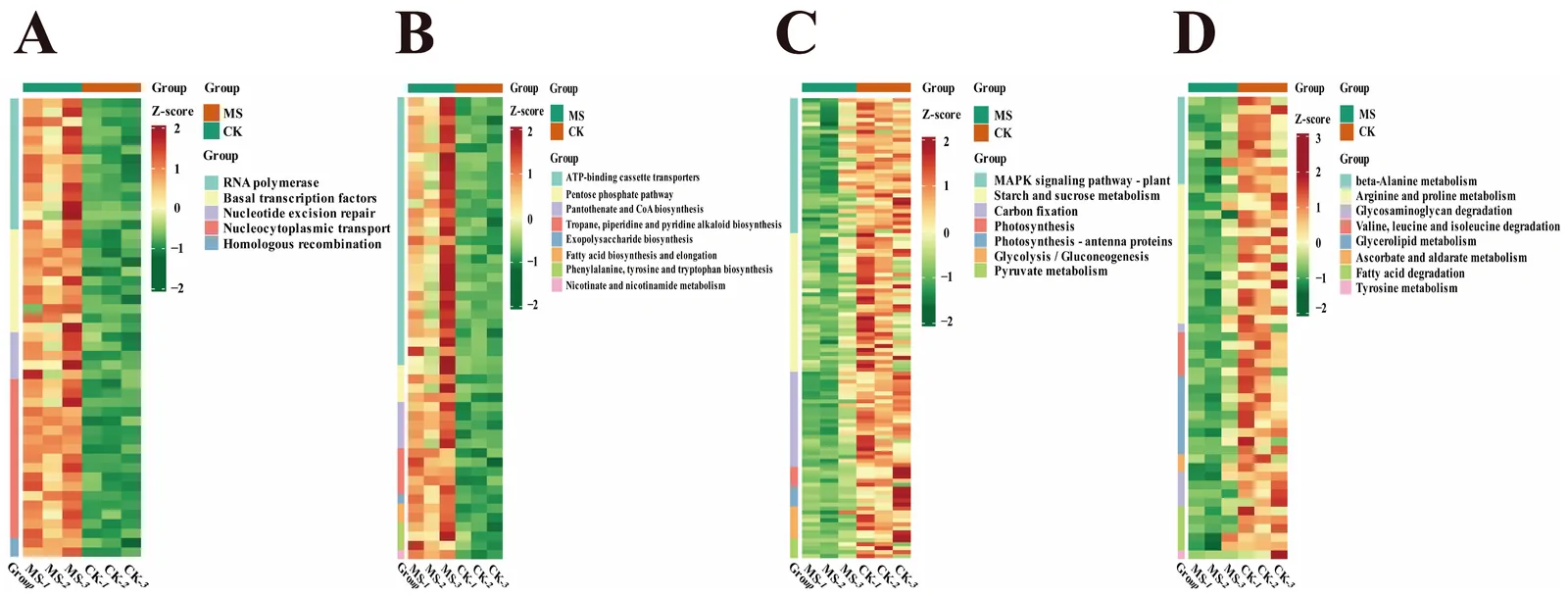
Heatmaps of representative key gene expression patterns in desert *Chlorella* sp. under moderate drought stress (MS vs. CK). (**A**–**D**) Heatmaps of representative stress-associated genes. CK: control; MS: moderate drought stress.

**Figure 9:**
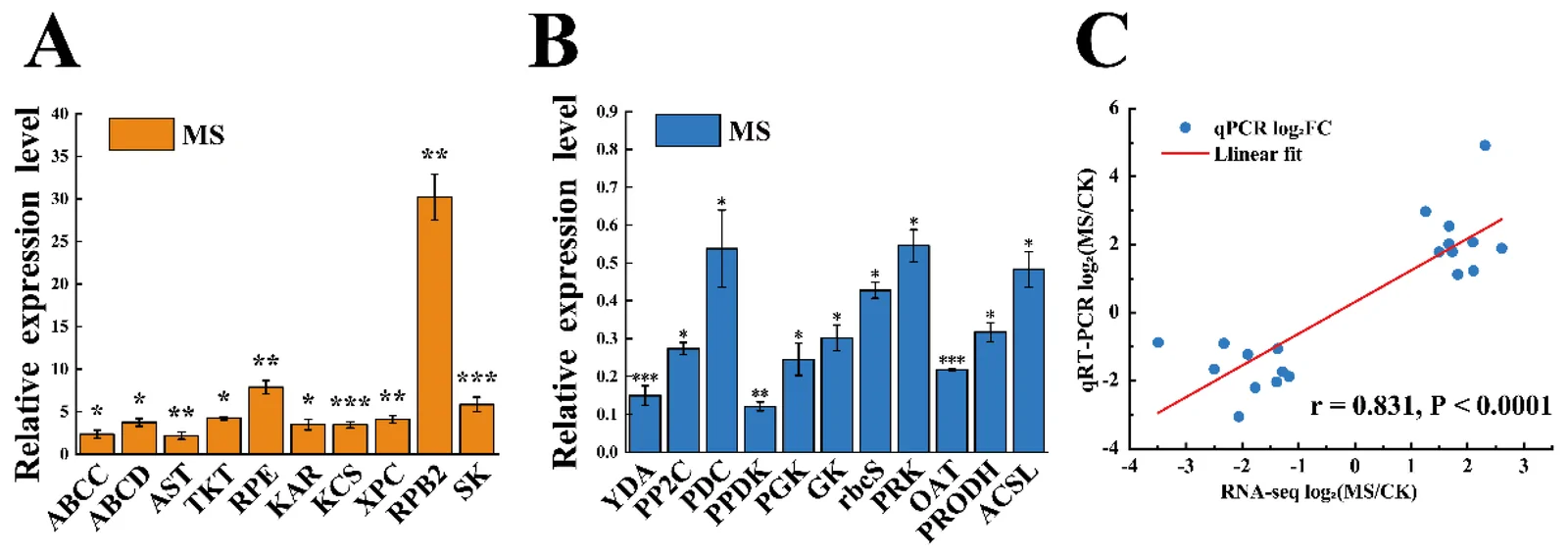
Validation of RNA-seq data by RT-qPCR in desert *Chlorella* sp. under moderate drought stress (MS vs. CK). (**A**) qRT-PCR validation for selected up-regulated genes; (**B**) qRT-PCR validation for selected down-regulated genes; (**C**) Pearson correlation analysis of log_2_(MS/CK) fold changes between RNA-seq and qRT-PCR for selected genes. CK: control; MS: moderate drought stress. * *p* < 0.05; ** *p* < 0.01; *** *p* < 0.001.

**Figure 10:**
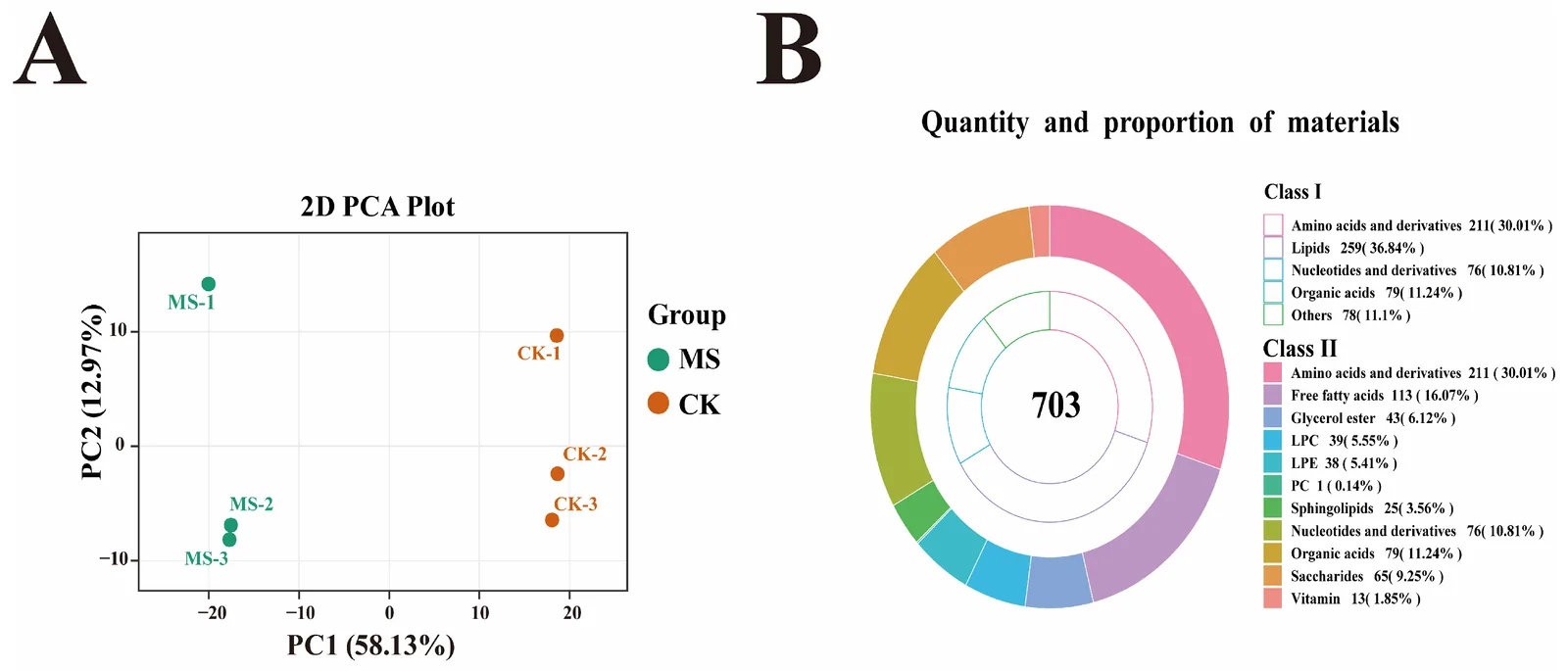
PCA and metabolite classification analysis of metabolomic changes in desert *Chlorella* sp. under moderate drought stress (MS vs. CK). (**A**) PCA score plot; (**B**) Classification and count of annotated metabolites, percentages may not sum to 100% due to rounding. CK: control; MS: moderate drought stress.

**Figure 11:**
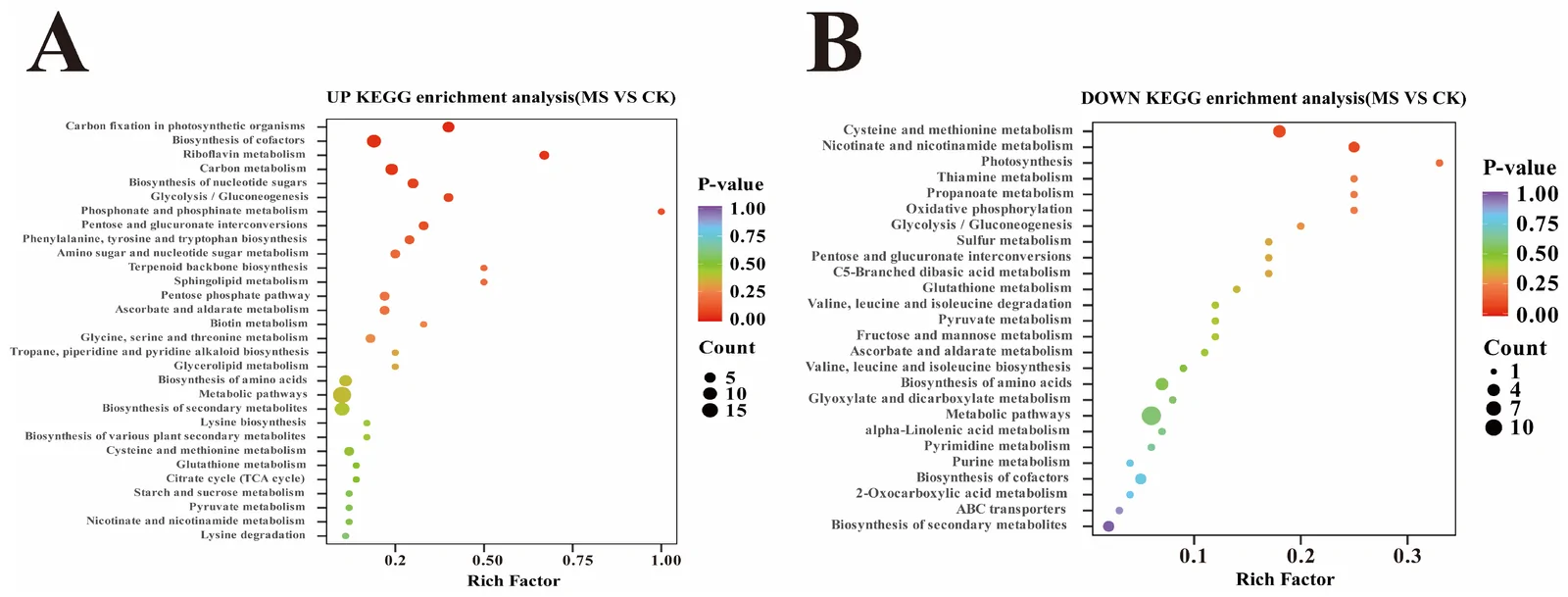
KEGG enrichment analysis of differential metabolites in desert *Chlorella* sp. under moderate drought stress (MS vs. CK). (**A**) KEGG enrichment scatter plot for metabolites with higher abundance in MS; (**B**) KEGG enrichment scatter plot for metabolites with lower abundance in MS. CK: control; MS: moderate drought stress.

**Table 1:** Relative abundance changes of representative differential metabolites under moderate drought stress.

Metabolites		UP	DOWN	Metabolites		UP	DOWN
Lipid				Amino Acid			
SP					Ser	3.165 ± 0.221 ***	
	d18:0	9.078 ± 0.584 **			Asn	3.361 ± 0.654 *	
FA					Lys	3.511 ± 0.033 ***	
	FA 14:0;13Me	2.311 ± 0.271 *			Arg	3.616 ± 0.353 **	
	FA 16:0;O2	7.464 ± 2.193 *			Trp	7.273 ± 2.505 *	
	FA 18:1;O	4.362 ± 0.095 ***			AcSpd		0.085 ***
	FA 22:2	2.075 ± 0.181 **			Leu		0.267 ± 0.032 **
	FA 20:4;O2	39.580 ± 1.090 ***			Thr		0.104 ***
	FA 18:3;O		0.119 ***		Val		0.168 ***
GLY					TPQ		0.232 ± 0.095 **
	MG 18:4	5.809 ± 0.698 **		Nucleotide			
	MG16:2		0.0253 ***		cAMP	4.213 ± 0.140 ***	
	MG16:3		0.139 ± 0.012 ***		2-AP	3.779 ± 0.213 **	
	MG 18:1		0.122 ± 0.003 ***		Ara-Hx	3.122 ± 0.181 **	
	MG 18:2		0.011 ± 0.006 ***		SAMP	2.911 ± 0.226 **	
	MGDG 18:3		0.0169 ***		Urd	2.629 ± 0.593 *	
LPC					NADP		0.285 ± 0.063 **
	LPC 18:3	5.941 ± 0.492 **			2-MeG		0.404 ± 0.030 ***
	LPC 17:1		0.145 ± 0.005 ***		2-MeOAdo		0.463 ± 0.089 **
	LPC 17:2		0.125 ± 0.003 ***	Organic Acid			
	LPC 18:0		0.162 ± 0.013 ***		GPA	2.879 ± 0.310 **	
	LPC 18:1		0.177 ± 0.017 ***		PEP	2.500 ± 0.280 *	
	LPC 19:0		0.164 ± 0.043 ***		Maleic acid	2.238 ± 0.262 *	
LPE					3-PGA	2.233 ± 0.202 **	
	LPE 14:0	3.695 ± 0.233 **			Leucic acid		0.363 ± 0.098 ***
	LPE 15:0	18.991 ± 1.454 **			HMG		0.496 ± 0.108 *
	LPE 16:0	2.859 ± 0.341 *			HMB		0.149 ± 0.010 **
	LPE 17:0	2.884 ± 0.148 **			OA		0.389 ± 0.105 *
	LPE 18:1	2.369 ± 0.423 *		Vitamin			
Sugars					NA	6.014 ± 0.538 **	
	Glc-1,6-P2	4.937 ± 1.084 *			INA	5.435 ± 0.430 **	
	GroPIns	3.924 ± 0.405 **			Bio	5.401 ± 0.402 **	
	GlcN-1P	3.801 ± 0.567 *			AA	2.285 ± 0.160 **	
	Tre	∞					
	D-Arabinonic acid		0.391 ± 0.043 ***				
	D-Xylonic acid		0.464 ± 0.059 **				
	Maltitol		0.249 ± 0.011 **				
	1,5-AG		0.036 ***				

* *p* < 0.05; ** *p* < 0.01; *** *p* < 0.001. ∞ indicates metabolite not detected within control samples. SD values were <0.00001 for all samples. Data are expressed as relative values (no units), with the control normalized to 1.0. Values are presented as mean ± SD (*n* = 3).

## Data Availability

Raw RNA-sequencing reads were deposited into NCBI Sequence Read Archive (SRA) under accession SRR39981469, SRR39981468, SRR39981467, SRR39981466, SRR39981465, SRR39981464. Processed metabolomic tables for core analysis are presented within the main manuscript. The metabolite abbreviation annotation table is provided in the Supplementary Materials. The unprocessed raw metabolite intensity matrix can be obtained from the corresponding author upon reasonable request.

## Supplementary Materials

The following supporting information can be downloaded at: https://www.mdpi.com/article/10.3390/ijms27198439/s1.

## Abbreviations

Key abbreviations used in this manuscript:

ABC	ATP-binding cassette
ABA	abscisic acid
CAT	catalase
DCW	dry cell weight
DEG	differentially expressed gene
ETR	electron-transport rate
EPS	exopolysaccharides
*Fv*/*Fm*	maximum quantum yield of photosystem II
JA	jasmonic acid
LSCM	laser-scanning confocal microscopy
MAPK	mitogen-activated protein kinase
MDA	malondialdehyde
OD	optical density
PCA	principal component analysis
PEG-6000	polyethylene glycol 6000
Pro	proline
qRT-PCR	quantitative real-time polymerase chain reaction
SEM	scanning electron microscopy
SOD	superoxide dismutase
TEM	transmission electron microscopy
ΦPSII	effective quantum yield of photosystem II
Y(NO)	non-regulated non-photochemical-dissipation yield

## References

[1] Thoré E.S.J., Muylaert K., Bertram M.G., Brodin T. (2023). Microalgae. Curr. Biol..

[2] Liu F., Jin S., Song H. (2026). Tructural Remodeling and Enzymatic Replacement Shape the Evolution of Organellar Group II Introns in *Ulva*. Int. J. Mol. Sci..

[3] Ramachandran S., Subramaniyam S., Simab K., Govindarajan R., Balasubramani R., Aran I. (2022). Microalgal Biorefinery Concepts’ Developments for Biofuel and Bioproducts: Current Perspective and Bottlenecks. Int. J. Mol. Sci..

[4] Barsanti L., Birindelli L., Gualtieri P. (2022). Paramylon and Other Bioactive Molecules in Micro and Macroalgae. Int. J. Mol. Sci..

[5] Wang Z., Tian J., Wang Y., Tang Y., Cui S. (2025). Structure-guided valorization of microalgal biomass for renewable and versatile applications. Bioresour. Technol..

[6] Tsintzou G., Bataka E., Tagkalaki G., Keisaris S., Tsiropoulos N., Labrou N., Madesis P. (2026). Biosynthesis of UV-Absorbing Mycosporine-like Amino Acids and Transcriptomic Profiling of Differential Gene Expression in Green Microalga Under Abiotic Stresses. Int. J. Mol. Sci..

[7] Suparmaniam U., Lam M.K., Lim J.W., Tan I.S., Chin B.L.F., Shuit S.H., Lim S., Pang Y.L., Kiew P.L. (2023). Abiotic stress as a dynamic strategy for enhancing high value phytochemicals in microalgae: Critical insights, challenges and future prospects. Biotechnol. Adv..

[8] Ryu Y.K., Lee W.K., Park G.H., Kim T., Lee Y., Koh E.J., Heo S.J., Choi W.Y., Oh C. (2024). Preliminary assessment of astaxanthin production in a new *Chlamydomonas* strain. Algal Res..

[9] Tan Z., Liu Y., Zhu Q., Bi J. (2022). Effect of dust aerosols on the heat exchange over the Taklimakan Desert. Atmos. Environ..

[10] Li M., Zhang Z., Ju C., Li H. (2023). Sensitivity of temperature and precipitation characteristics to land use classification over the Taklimakan Desert and surrounding area. Theor. Appl. Climatol..

[11] Zhang W., Ye G., Leung J.C.H., Zhang B. (2025). Microphysical characteristics of summer precipitation over the Taklamakan Desert based on GPM-DPR data from 2014 to 2023. Atmosphere.

[12] Zhang B.C., Zhang Y.M., Zhao J.C., Wu N., Chen R.Y., Zhang J. (2009). Microalgal species variation at different successional stages in biological soil crusts of the Gurbantunggut Desert, northwestern China. Biol. Fertil. Soils.

[13] Bhakat S., Mondal A., Mandal S., Rath J. (2024). Role of exopolysaccharides of *Anabaena* sp. in desiccation tolerance and biodeterioration of ancient terracotta monuments of Bishnupur. Arch. Microbiol..

[14] Maurya N., Sharma A., Agrawal M., Sundaram S. (2025). Role of novel *Chlorella vulgaris* SSAU8 in improving the soil health under induced drought stress. Curr. Microbiol..

[15] Shetty P., Gitau M.M., Maróti G. (2019). Salinity stress responses and adaptation mechanisms in eukaryotic green microalgae. Cells.

[16] Dabravolski S.A., Isayenkov S.V. (2022). Metabolites facilitating adaptation of desert cyanobacteria to extremely arid environments. Plants.

[17] Li C., Chen Z., Chen L., Wang G. (2025). The adaptation mechanism of desert soil cyanobacterium *Chroococcidiopsis* sp. to desiccation. Plant Physiol. Biochem..

[18] Shang J.L., Xie Y.X., Shi L.Y., Diao S.R., Guan J.Y. (2025). Molecular mechanisms of *Nostoc flagelliforme* environmental adaptation: A comprehensive review. Plants.

[19] Aigner S., Glaser K., Arc E., Holzinger A., Schletter M., Karsten U., Kranner I. (2020). Adaptation to aquatic and terrestrial environments in *Chlorella vulgaris* (Chlorophyta). Front. Microbiol..

[20] Bosch J., Varliero G., Hallsworth J.E., Dallas T.D., Hopkins D., Frey B., Kong W., Lebre P., Makhalanyane T.P., Cowan D.A. (2021). Microbial anhydrobiosis. Environ. Microbiol..

[21] Sun Y., Wang C., Chen H.Y.H., Ruan H. (2020). Response of plants to water stress: A meta-analysis. Front. Plant Sci..

[22] Song F., Yang Q., Huang J., Guo Z., Li Y., Deng W. (2026). Plant drought stress: Physiological, biochemical and molecular mechanisms. Plant Stress.

[23] Rajendran U.M., Kathirvel E., Anand N. (2007). Desiccation-induced changes in antioxidant enzymes, fatty acids, and amino acids in the cyanobacterium *Tolypothrix scytonemoides*. World J. Microbiol. Biotechnol..

[24] Hell A.F., Gasulla F., González-Hourcade M., Del Campo E.M., Centeno D.C., Casano L.M. (2019). Tolerance to cyclic desiccation in lichen microalgae is related to habitat preference and involves specific priming of the antioxidant system. Plant Cell Physiol..

[25] Tafvizi F., Hosseini Tafreshi S.A., Toluei Z., Toghyani M.A. (2020). Stress responses of the green microalga, *Dunaliella salina* to PEG-induced drought. J. Mar. Biol. Assoc. U.K..

[26] Han K., Zhou Y., Zhang K., Wang L., Gao J., Chen F. (2024). Evaluation of drought resistance in three *Chlorella* strains. Shengwu Jishu Tongbao.

[27] Hosseini Tafreshi S.A., Aghaie P., Toghyani M.A., Ramazani-Moghaddam-Arani A. (2020). Improvement of ionizing gamma irradiation tolerance of *Chlorella vulgaris* by pretreatment with polyethylene glycol. Int. J. Radiat. Biol..

[28] Claeys H., Skirycz A., Maleux K., Inzé D. (2012). DELLA signaling mediates stress-induced cell differentiation in *Arabidopsis* leaves through modulation of anaphase-promoting complex/cyclosome activity. Plant Physiol..

[29] Holzinger A., Karsten U. (2013). Desiccation stress and tolerance in green algae: Consequences for ultrastructure, physiological and molecular mechanisms. Front. Plant Sci..

[30] González-Hourcade M., Braga M.R., Del Campo E.M., Ascaso C., Patiño C., Casano L.M. (2020). Ultrastructural and biochemical analyses reveal cell wall remodelling in lichen-forming microalgae submitted to cyclic desiccation-rehydration. Ann. Bot..

[31] Tang S., Liu Y., Zhu J., Cheng X., Liu L., Hammerschmidt K., Zhou J., Cai Z. (2024). Bet hedging in a unicellular microalga. Nat. Commun..

[32] Shiratake T., Sato A., Minoda A., Tsuzuki M., Sato N. (2013). Air-drying of cells, the novel conditions for stimulated synthesis of triacylglycerol in a green alga, *Chlorella kessleri*. PLoS ONE.

[33] Gamboa-Tuz S.D., Pereira-Santana A., Zamora-Briseño J.A., Castano E., Espadas-Gil F., Ayala-Sumuano J.T., Keb-Llanes M.Á., Sanchez-Teyer F., Rodríguez-Zapata L.C. (2018). Transcriptomics and coexpression networks reveal tissue-specific responses and regulatory hubs under mild and severe drought in papaya *Carica papaya* L.. Sci. Rep..

[34] Colina F.J., Carbó M., Cañal M.J., Valledor L. (2020). A complex metabolic rearrangement towards the accumulation of glycerol and sugars consequence of a proteome remodeling is required for the survival of *Chlamydomonas reinhardtii* growing under osmotic stress. Environ. Exp. Bot..

[35] Wang B., Li X., Wang G. (2023). Responses of the desert green algae, *Chlorella* sp. to drought stress. J. Phycol..

[36] Sharma P., Choudhury S., Rout J. (2026). Desiccation-induced oxidative stress causes metabolic flux in the terrestrial green alga *Trentepohlia* sp., suggesting extensive metabolic reprogramming for stress adaptation. Appl. Biochem. Biotechnol..

[37] Ingrisano R., Tosato E., Trost P., Gurrieri L., Sparla F. (2023). Proline, cysteine and branched-chain amino acids in abiotic stress response of land plants and microalgae. Plants.

[38] Xiong J., Ma N. (2022). Transcriptomic and metabolomic analyses reveal that fullerol improves drought tolerance in *Brassica napus* L.. Int. J. Mol. Sci..

[39] Li X., Liu Y., Hu W., Yin B., Liang B., Li Z., Zhang X., Xu J., Zhou S. (2024). Integrative physiological, metabolomic, and transcriptomic analysis reveals the drought responses of two apple rootstock cultivars. BMC Plant Biol..

[40] Rodas-Junco B.A., Racagni-Di-Palma G.E., Canul-Chan M., Usorach J., Hernández-Sotomayor S.M.T. (2021). Link between lipid second messengers and osmotic stress in plants. Int. J. Mol. Sci..

[41] Cahoon E.B., Li-Beisson Y. (2020). Plant unusual fatty acids: Learning from the less common. Curr. Opin. Plant Biol..

[42] Fujiwara Y., Hama K., Shimozawa N., Yokoyama K. (2021). Glycosphingolipids with very long-chain fatty acids accumulate in fibroblasts from adrenoleukodystrophy patients. Int. J. Mol. Sci..

[43] Hao T.B., Lai P.Y., Shu Z., Liang R., Chen Z.Y., Huang R.L., Lu Y., Alimujiang A. (2025). Physiological and metabolic fluctuations of the diatom *Phaeodactylum tricornutum* under water scarcity. Front. Microbiol..

[44] Song L., Jian L., Yan B., Deng X., Qing L., Pu Y., Huang F., Jiao Z., Liu S., Zhou M. (2024). The transcriptomic and metabolomic profiles of *E. californica* revealed pathways related to drought stress response and close association with alkaloid biosynthesis. Ind. Crops Prod..

[45] Liao S., Li K., Wei Y., Zhao S., Zhang M., Wang J., Jiang J., Chen T., Wu F., Fan J. (2025). Nucleobase-ascorbate transporter OsNAT9 regulates seed vigor and drought tolerance by modulating ascorbic acid homeostasis in rice. Plant J..

[46] Zhi X., Bian X., Yu J., Xiao X., Duan B., Huang F., Jiang Z., Zhou G., Ma N. (2024). Comparative metabolomics analysis of tolerant and sensitive genotypes of rapeseed (*Brassica napus* L.) seedlings under drought stress. Agric. Water Manag..

[47] Huang X., Rong W., Zhang X., Gao Y., Zhou Y., Su J., Luo H., Chu G., Wang M. (2024). Transcriptome and metabolome analysis reveal the dynamic changes and biosynthesis pathways of alkaloids in *Sophora alopecuroides* L. under drought stress. Ind. Crops Prod..

[48] Jawad H.M., Hongyin Q., Bizhen C., Shafiq H., Yan P., Wei L., Guangyan F., Junming Z., Zhou L. (2022). Enhanced adaptability to limited water supply regulated by diethyl aminoethyl hexanoate (DA-6) associated with lipidomic reprogramming in two white clover genotypes. Front. Plant Sci..

[49] Zheng Y., Xia Z., Wu J., Ma H. (2021). Effects of repeated drought stress on the physiological characteristics and lipid metabolism of *Bombax ceiba* L. during subsequent drought and heat stresses. BMC Plant Biol..

[50] Gong X., Wang S. (2022). New insights into evolution of the ABC transporter family in *Mesostigma viride*, a unicellular charophyte algae. Curr. Issues Mol. Biol..

[51] Khasin M., Cahoon R.E., Alvarez S., Beckeris R.G., Riekhof W.R. (2017). Synthesis, secretion, and perception of abscisic acid regulates stress responses in *Chlorella sorokiniana*. bioRxiv.

[52] Bai L., Wang Y., Zhou X., Liu Q., Dong L., Che Z., Zhou Z., Wang Y. (2025). Exogenous methyl jasmonate activates the jasmonic acid signaling pathway in *Chlorella* sp. to alleviate 2,2′,4,4′-tetrabromodiphenyl ether stress. J. Phycol..

[53] Li Y., Feng D., Zhang D., Su J., Zhang Y., Li Z., Mu P., Liu B., Wang H., Wang J. (2012). Rice MAPK phosphatase IBR5 negatively regulates drought stress tolerance in transgenic *Nicotiana tabacum*. Plant Sci..

[54] Liu Y., Yu T., Li Y., Zheng L., Lu Z., Zhou Y., Chen J., Chen M., Zhang J., Sun G. (2022). Mitogen-activated protein kinase TaMPK3 suppresses ABA response by destabilising TaPYL4 receptor in wheat. New Phytol..

[55] Yamada K. (2026). No energy, no defense: Metabolic input shapes defense signaling. Curr. Opin. Plant Biol..

[56] Wang D., Gong C., Gou Y., Zhou L., Zhu J., Gao J. (2014). Phylogenetic analysis of several algae in biological soil crusts of the Taklamakan Desert. Caoye Xuebao.

[57] Badr A.A., Fouad W.M. (2021). Identification of culturable microalgae diversity in the River Nile in Egypt using enrichment media. Afr. J. Biol. Sci..

[58] Wang W., Wu X., Tan J., Zhu L., Mou Y., Zhang D., Gao J. (2018). Using response surface methodology to optimize culture conditions for human lactoferrin production in desert *Chlorella*. Protein Expr. Purif..

[59] Akdeniz B., Wood J.A., Lammertink R.G.H. (2024). Diffusiophoresis in polymer and nanoparticle gradients. J. Phys. Chem. B.

[60] Berestovsky G.N., Ternovsky V.I., Kataev A.A. (2001). Through pore diameter in the cell wall of *Chara corallina*. J. Exp. Bot..

[61] Wakabayashi K., Shibatsugu M., Hattori T., Soga K., Hoson T. (2025). Mechanisms involved in cell wall remodeling in etiolated rice shoots grown under osmotic stress. Life.

[62] Cherepanov D.A., Petrova A.A., Fadeeva M.S., Gostev F.E., Shelaev I.V., Nadtochenko V.A., Semenov A.Y. (2024). Specificity of photochemical energy conversion in photosystem I from the green microalga *Chlorella ohadii*. Biochemistry.

[63] Morris D.L. (1948). Quantitative determination of carbohydrates with Dreywood’s anthrone reagent. Science.

[64] Epstein E., Baginski E.S., Zak B. (1972). Extraction of lipids from serum and measurement of total serum lipids. Ann. Clin. Lab. Sci..

[65] Bradford M.M. (1976). A rapid and sensitive method for the quantitation of microgram quantities of protein utilizing the principle of protein-dye binding. Anal. Biochem..

[66] Bates L.S., Waldren R.P., Teare I.D. (1973). Rapid determination of free proline for water-stress studies. Plant Soil.

[67] Schmedes A., Hølmer G. (1989). A new thiobarbituric acid (TBA) method for determining free malondialdehyde (MDA) and hydroperoxides selectively as a measure of lipid peroxidation. J. Am. Oil Chem. Soc..

[68] Beers R.F., Sizer I.W. (1952). A spectrophotometric method for measuring the breakdown of hydrogen peroxide by catalase. J. Biol. Chem..

[69] Beauchamp C., Fridovich I. (1971). Superoxide dismutase: Improved assays and an assay applicable to acrylamide gels. Anal. Biochem..

[70] Zhang K., Wei Z., Wang Y., Shui W., Jia B., Huang Z., Feng Q. (2025). Green under siege: How the polyhexamethylene guanidine disinfectant attacks microalgae in aquatic ecosystems. PeerJ.

[71] Ritchie R.J. (2008). Universal chlorophyll equations for estimating chlorophylls a, b, c, and d and total chlorophylls in natural assemblages of photosynthetic organisms using acetone, methanol, or ethanol solvents. Photosynthetica.

[72] Bordenave C.D., García-Breijo F., Gazquez A., Muggia L., Carrasco P., Barreno E. (2023). Low temperature scanning electron microscopy (LTSEM) findings on the ultrastructure of *Trebouxia lynnae* (Trebouxiophyceae, lichenized microalgae). Diversity.

[73] Xie H., Cheng H.S., Ng J.X.J., Zhang S., Kim J.H.S., Tan S.H., Tan C.H., Tan N.S. (2026). A gut-liver lipid flux checkpoint mediates FAHFA protection from MASLD. Pharmacol. Res..

[74] Liu D., Yang W., Lv Y., Li S., Qv M., Dai D., Zhu L. (2023). Pollutant removal and toxic response mechanisms of freshwater microalgae *Chlorella sorokiniana* under exposure of tetrabromobisphenol A and cadmium. Chem. Eng. J..

[75] Aigner S., Arc E., Schletter M., Karsten U., Holzinger A., Kranner I. (2022). Metabolite profiling in green microalgae with varying degrees of desiccation tolerance. Microorganisms.

