# Supplementary material for: Physiological–Biochemical Characteristics and Omics Landscape of Drought Tolerance in Chlorella sp. from the Taklamakan Desert of China
Source: Int J Mol Sci. 2026 Sep 22;27(19):8439. doi: 10.3390/ijms27198439 (PMC13664879; doi:10.3390/ijms27198439)
Supplement: Supplementary file 1 [file ijms-27-08439-s001.zip › Figures S10.pdf]

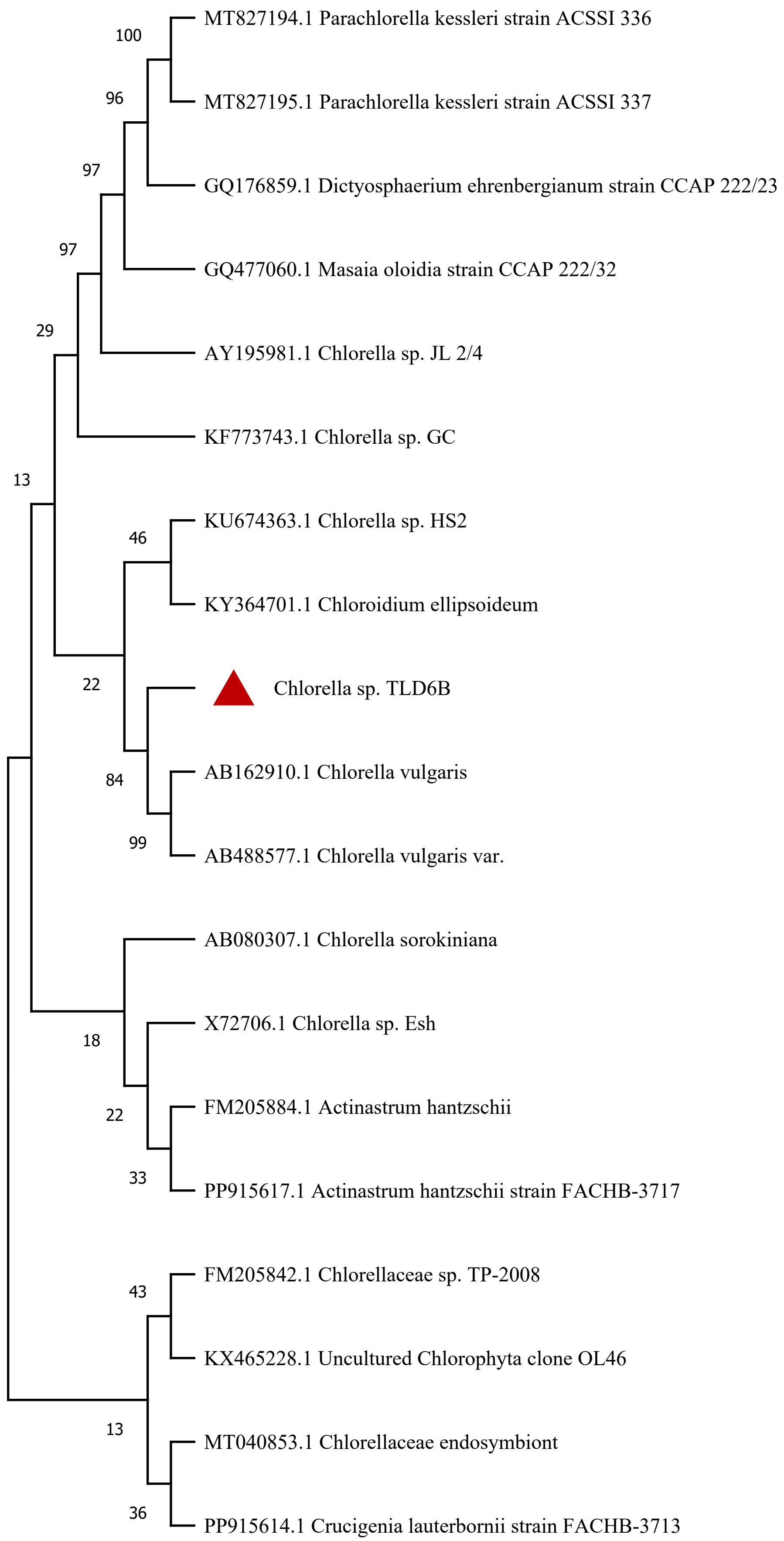
MT827194.1 Parachlorella kessleri strain ACSSI 336
MT827195.1 Parachlorella kessleri strain ACSSI 337
GQ176859.1 Dictyosphaerium ehrenbergianum strain CCAP 222/23
GQ477060.1 Masaia oloidia strain CCAP 222/32
AY195981.1 Chlorella sp. JL 2/4
KF773743.1 Chlorella sp. GC
KU674363.1 Chlorella sp. HS2
KY364701.1 Chloroidium ellipsoideum
Chlorella sp. TLD6B
AB162910.1 Chlorella vulgaris
AB488577.1 Chlorella vulgaris var.
AB080307.1 Chlorella sorokiniana
X72706.1 Chlorella sp. Esh
FM205884.1 Actinastrum hantzschii
PP915617.1 Actinastrum hantzschii strain FACHB-3717
FM205842.1 Chlorellaceae sp. TP-2008
KX465228.1 Uncultured Chlorophyta clone OL46
MT040853.1 Chlorellaceae endosymbiont
PP915614.1 Crucigenia lauterbornii strain FACHB-3713
100
96
97
97
29
13
46
22
84
99
18
22
33
43
13
36
