# Supplementary material for: Physiological–Biochemical Characteristics and Omics Landscape of Drought Tolerance in Chlorella sp. from the Taklamakan Desert of China
Source: Int J Mol Sci. 2026 Sep 22;27(19):8439. doi: 10.3390/ijms27198439 (PMC13664879; doi:10.3390/ijms27198439)
Supplement: Supplementary file 1 [file ijms-27-08439-s001.zip › ijms-4588548-supplementary captions.pdf]

1. Supplementary File S1: Culture Conditions.

2. Supplementary File S2: Ultrastructural Observation Methods.

3. Supplementary File S3: Transcriptomic Analysis.

4. Supplementary File S4: Metabolomic Analysis.

5. Supplementary Table S1: List of significantly up-regulated genes.

6. Supplementary Table S2: List of significantly down-regulated genes.

7. Supplementary Table S3: qRT-PCR target-gene information and primer sequences.

8. Supplementary Table S4: Metabolite-abbreviation cross-reference table.

9. Figure S1: Transcriptome biological replicate correlation heatmap.

10. Figure S2: KEGG pathway enrichment of down-regulated genes.

11. Figure S3: KEGG pathway enrichment of up-regulated genes.

12. FigureS4: Widely targeted metabolomics biological replicate correlation heatmap.

13. Figure S5: Classification of up-regulated metabolites.

14. Figure S6: Classification of down-regulated metabolites.

15. Figure S7: KEGG pathway enrichment of up-regulated metabolites.

16. Figure S8: KEGG pathway enrichment of down-regulated metabolites.

17. FigureS9: Quality control assessments for targeted sugar and organic acid metabolomic datasets.

18. Figure S10: Phylogenetic placement of Chlorella sp. TLD 6B inferred from 18S rRNA gene sequences. The 18S rRNA sequence of strain TLD 6B is provided as Supplementary File S5.
