# Supplementary material for: Physiological–Biochemical Characteristics and Omics Landscape of Drought Tolerance in Chlorella sp. from the Taklamakan Desert of China
Source: Int J Mol Sci. 2026 Sep 22;27(19):8439. doi: 10.3390/ijms27198439 (PMC13664879; doi:10.3390/ijms27198439)
Supplement: Supplementary file 1 [file ijms-27-08439-s001.zip › Supplementary File S1 Culture Conditions.pdf]

Culture conditions: Cultures were maintained at 25 °C under a 12 h light / 12 h dark photoperiod. Illumination was provided by cool-white LED grow lights at an intensity of 6000 lx. Cultures were grown in 150 mL glass Erlenmeyer flasks with an inoculation volume of 100 mL. Prior to inoculation, both the culture vessels and the medium were autoclaved at 121 °C and 0.1 MPa for 20 min. The cultures were maintained under static conditions with gentle manual agitation once daily, and all flasks were kept at the same distance from the light source.

Main reagents and chemicals:

All reagents used in this study were of analytical grade and purchased from Sinopharm Chemical Reagent Co., Ltd. (Shanghai, China). The reagents and their CAS registry numbers are listed below

Reagent	CAS No.
Zinc sulfate heptahydrate ($ZnSO_4 \cdot 7H_2O$)	7446-20-0
Manganese chloride tetrahydrate ($MnCl_2 \cdot 4H_2O$)	13446-34-9
Cobalt nitrate hexahydrate ($Co(NO_3)_2 \cdot 6H_2O$)	10026-22-9
Molybdenum trioxide (MoO_3)	1313-27-5
Copper sulfate pentahydrate ($CuSO_4 \cdot 5H_2O$)	7758-99-8
Boric acid (H_3BO_3)	10043-35-3
Iron(II) sulfate heptahydrate ($FeSO_4 \cdot 7H_2O$)	7782-63-0
Ethylenediaminetetraacetic acid (EDTA)	60-00-4
Potassium hydroxide (KOH)	1310-58-3
Sodium chloride (NaCl)	7647-14-5
Potassium dihydrogen phosphate (KH_2PO_4)	7778-77-0
Calcium chloride dihydrate ($CaCl_2 \cdot 2H_2O$)	10035-04-8
Dipotassium hydrogen phosphate (K_2HPO_4)	7758-11-4
Magnesium sulfate heptahydrate ($MgSO_4 \cdot 7H_2O$)	10034-99-8
Sodium nitrate ($NaNO_3$)	7631-99-4

Composition of the 1000× stock solution of BBM (Bold's Basal Medium)x1000（BBM）:

Component	Weight	Component	Weight
$ZnSO_4 \cdot 7H_2O$	0.882g	$CuSO_4 \cdot 5H_2O$	0.157g
$MnCl_2 \cdot 7H_2O$	0.121g	$FeSO_4 \cdot 7H_2O$	0.498g
Co(NO3)$_2 \cdot 6H_2O$	0.049g	KH_2PO_4	17.5g
MoO	0.071g	$CaCl_2 \cdot 2H_2O$	2.5g
H_3BO_3	1.142g	K_2HPO_4	7.5g
EDTA	5g	$MgSO_4 \cdot 7H_2O$	7.5g
KOH	3.1g	$NaNO_3$	25g
NaCl	2.5g		
