# Supplementary material for: Physiological–Biochemical Characteristics and Omics Landscape of Drought Tolerance in Chlorella sp. from the Taklamakan Desert of China
Source: Int J Mol Sci. 2026 Sep 22;27(19):8439. doi: 10.3390/ijms27198439 (PMC13664879; doi:10.3390/ijms27198439)
Supplement: Supplementary file 1 [file ijms-27-08439-s001.zip › Supplementary File S2 Ultrastructural Observation Methods.pdf]

Sample preparation for scanning electron microscopy (SEM)

(1) Sample processing and fixation: Algal pellets (0.5 g) from both MS-treated and CK-treated groups were collected in triplicate. The pellets were washed three times with phosphate-buffered saline (PBS) by centrifugation at 8,000 × g for 5 min at room temperature to remove residual culture medium and impurities. After discarding the supernatant, the pelleted cells were completely submerged in 2.5% glutaraldehyde fixative (electron microscopy grade) and allowed to stand at room temperature for 2 h, followed by overnight fixation at 4 °C in the dark for 12–18 h.

(2) Washing and gradient dehydration: The fixed samples were gently rinsed three times with PBS on an orbital shaker, 15 min for each wash. Subsequently, the samples were sequentially immersed in pre-chilled ethanol gradients of 30%, 50%, 70%, 80%, 90%, 95%, and 100% for dehydration, with 15 min incubation at each concentration. Each dehydration step was followed by centrifugation at 8,000 rpm for 5 min at 4 °C.

(3) Drying, mounting, and gold coating: The dehydrated samples were thoroughly dried using a critical point dryer with liquid CO_2 as the displacement medium, yielding a fluffy algal powder. A small amount of the dried algal powder was evenly attached onto aluminum specimen stubs (SEM stubs) using double-sided conductive carbon tape, and loosely adhered powder was gently blown off with a rubber bulb. The samples were then coated with gold using an ion sputter coater to enhance conductivity, with a sputtering duration of 60–90 s at a current of 20 mA.

(4) SEM observation: The prepared specimen stubs were securely placed into the chamber of a field-emission scanning electron microscope (FE-SEM). The observation parameters were set as follows: working distance (WD) of 6–8 mm, accelerating voltage of 2.0 kV, and emission current of 10 μA. Images were captured using a secondary electron (SE) detector at magnifications of ×3,000 (medium), ×4,000 (high), and ×20,000 (ultra-high).

Sample preparation for transmission electron microscopy (TEM)

(1) Sample processing, prefixation, washing, and postfixation: The procedures for sample processing, prefixation, and washing were identical to those described in steps (1) and (2) of the SEM sample preparation. After washing, the algal pellets were immersed in 1% osmium tetroxide (OsO_4) fixative and fixed at room temperature in the dark for 4 h. Subsequently, the samples were rinsed three times with PBS using the same centrifugation method to thoroughly remove residual osmium tetroxide.

(2) Gradient dehydration and solvent transition: Dehydration was performed using a graded series of pre-chilled ethanol solutions at concentrations of 30%, 50%, 70%, 80%, 90%, 95%, and 100% (with the 100% step repeated once), with 15 min incubation at each concentration. After dehydration, the ethanol in the samples was replaced with anhydrous acetone twice at room temperature, 15 min each time (centrifugation at 8,000 rpm for 5 min at room temperature).

(3) Infiltration, embedding, and polymerization: The dehydrated samples were immersed in a 1:1 (v/v) mixture of acetone and Epon 812 embedding resin and placed on a shaker at room temperature for slow infiltration (approximately 12 h). The samples were then transferred into pure Epon 812 embedding resin and infiltrated for an additional 6 h under the same conditions. After thorough infiltration, the samples were transferred into embedding capsules and polymerized in a constant-temperature oven at 60 °C for 48 h until the blocks reached an appropriate hardness.

(4) Ultrathin sectioning and staining: Ultrathin sections of 70–90 nm in thickness were cut from the embedding blocks using an ultramicrotome and collected on copper grids. The sections were sequentially stained with uranyl acetate solution for 20 min and lead citrate

solution for 10 min. After staining, the copper grids were gently rinsed three times with distilled water, blotted dry with filter paper, and dried in a desiccator.

(5) TEM observation: The copper grids bearing the sample sections were mounted onto a specimen holder and observed under a field-emission transmission electron microscope (FE-TEM). The observation parameters were set as follows: accelerating voltage of 80 kV and objective aperture of 40 μm. Special attention was paid to changes in the number and volume of starch grains and lipid droplets. Images were captured at magnifications of ×3,000 and ×4,000 (medium), ×7,000 and ×8,000 (high), and ×12,000 (ultra-high).

Sample preparation for laser scanning confocal microscopy (LSCM)

(1) Preparation of BODIPY 500/510 C_1, C_{12} stock and working solutions: BODIPY 500/510 C_1, C_{12} (0.01 g) was accurately weighed and dissolved in 2.47 mL of dimethyl sulfoxide (DMSO) with vortexing until completely dissolved, yielding a 10 mM stock solution, which was stored at −80 °C in the dark for up to 6 months. An aliquot of 10 μL of the stock solution was diluted with PBS to a final volume of 10 mL to obtain a 10 μM working solution, which was prepared freshly before use.

(2) Algal cell staining: Algal pellets (0.1 g) from both MS-treated and CK-treated groups were collected in triplicate. The pellets were washed three times with PBS by centrifugation at 8,000 rpm for 5 min at room temperature to remove residual culture medium and impurities. The washed pellets were then incubated with 1 mL of the freshly prepared BODIPY 500/510 C_1, C_{12} working solution at room temperature for 30 min. Subsequently, the samples were rinsed twice with PBS using the same centrifugation method. Finally, the stained cells were resuspended in PBS and adjusted to a cell density of approximately 1×10^6 cells/mL.

(3) LSCM observation: An aliquot of 1 mL of the stained cell suspension was transferred onto a glass slide and gently covered with a coverslip using forceps. The sample was then observed under a laser scanning confocal microscope equipped with a 60×/1.40 NA oil-immersion objective. BODIPY 500/510 C_1, C_{12} (lipid droplet fluorescence) was excited by a 488 nm argon ion laser, and the emission was collected in the range of 500–550 nm. Chlorophyll autofluorescence was excited by a 561 nm laser, with emission collected in the range of 650–750 nm. Bright-field images were simultaneously acquired using the transmitted light channel. All images were captured at a resolution of 1024 × 1024 pixels, with the pinhole set to 1 Airy Unit.

Image Acquisition and Statistical Analysis

For each treatment group (MS and CK), three independent biological replicates were set up. In each replicate, approximately 10 images at various magnifications were randomly acquired for SEM and TEM observations, and approximately 2 sets of different fluorescence images were acquired for LSCM. Representative images were selected for presentation. Image analysis and quantification were performed using the online software ImageJ.JS(https://ij.imjoy.io). Cell diameter (CD) was calculated from SEM images, and the normalized fluorescence intensity (NIF) of lipid droplets per unit area was determined from LSCM images. All data are expressed as means ± standard deviation (SD). Statistical analyses were performed using IBM SPSS Statistics 26.0 (IBM, Armonk, NY, USA). Tukey's post-hoc test with a significance level of $p < 0.05$ was used to determine significant differences among samples.

Main reagents and chemicals：

Reagent	Chemical formula	CAS No.	Specification	Source
Potassium chloride	KCl	7447-40-7	AR	Sinopharm, Shanghai, China
Glutaraldehyde	$C_5H_8O_2$	111-30-8	50% aq., EM	Sinopharm, Shanghai, China
Osmium tetroxide	OsO_4	20816-12-0	AR	Sinopharm, Shanghai, China
Acetone	C_3H_6O	67-64-1	AR	Sinopharm, Shanghai, China
Dimethyl sulfoxide (DMSO)	C_2H_6OS	67-68-5	AR	Aladdin, Shanghai, China
Isoamyl acetate	$C_7H_{14}O_2$	123-92-2	AR	GCRF, Guangzhou, China
Disodium hydrogen phosphate dihydrate	$Na_2HPO_4 \cdot 2H_2O$	10028-24-7	AR	Xinbote, Tianjin, China
Sodium dihydrogen phosphate monohydrate	$NaH_2PO_4 \cdot H_2O$	10049-21-5	AR	Xinbote, Tianjin, China
Potassium dihydrogen phosphate	KH_2PO_4	7778-77-0	AR	Sinopharm, Shanghai, China
Ethanol	C_2H_5OH	64-17-5	AR	Sinopharm, Shanghai, China
Sodium chloride	$NaCl$	7647-14-5	AR	Sinopharm, Shanghai, China
Uranyl acetate dihydrate	$C_4H_6O_6U \cdot 2H_2O$	5809-04-1	≥99.0%	Yaso, Linyi, China
Trisodium citrate dihydrate	$Na_3C_6H_5O_7 \cdot 2H_2O$	6106-32-7	AR	Aladdin, Shanghai, China
Lead nitrate	$Pb(NO_3)_2$	10099-74-8	AR	GCRF, Guangzhou, China
Sodium hydroxide	$NaOH$	1310-73-2	AR	Sinopharm, Shanghai, China
EMbed 812 embedding kit	–	–	–	Haide, Beijing, China
BODIPY 500/510 C_1, C_{12}	–	–	–	MedChemExpress, Monmouth Junction, NJ, USA

Preparation of Main Reagents

1. Phosphate-buffered saline (PBS, pH 7.4): KCl (0.2 g), NaCl (8 g), $Na_2HPO_4 \cdot 2H_2O$ (1.805 g), and KH_2PO_4 (0.24 g) were accurately weighed, dissolved in distilled water, and the volume was adjusted to 1000 mL. The pH was adjusted to 7.4.

2. Phosphate buffer (PB, pH 7.4): $Na_2HPO_4 \cdot 2H_2O$ (17.8 g) and $NaH_2PO_4 \cdot H_2O$ (15.6 g) were accurately weighed, dissolved in distilled water, and the volume was adjusted to 1000 mL. The pH was adjusted to 7.4.

3. Glutaraldehyde fixative (2.5%): Glutaraldehyde (25%, 10 mL) was accurately measured and diluted with PB to a final volume of 100 mL.

4. 1% Osmium tetroxide fixative: Osmium tetroxide (1 g) was accurately weighed in a fume hood, dissolved in PB (100 mL), and allowed to stand at 4 °C in the dark for 12 h with intermittent shaking every 2 h until completely dissolved.

5.Graded ethanol dehydration solutions: Ethanol (30, 50, 70, 80, 90, and 95 mL, respectively) was accurately measured and diluted with distilled water to a final volume of 100 mL for each concentration.

6. 1 M Sodium hydroxide solution: NaOH (4 g) was accurately weighed, dissolved in 80 mL of distilled water with stirring until completely dissolved, and then the volume was

adjusted to 100 mL with distilled water.

7. Uranyl acetate staining solution: Uranyl acetate powder (2 g) was accurately weighed, dissolved in 80 mL of distilled water with stirring until completely dissolved, and then the volume was adjusted to 100 mL with distilled water. The solution was filtered through a 0.22 μm membrane filter and stored at 4 °C in the dark.

8. Lead citrate staining solution: Lead nitrate (2.66 g) and trisodium citrate dihydrate (3.52 g) were accurately weighed and dissolved in 50 mL of distilled water with vigorous shaking for 2 min, followed by intermittent gentle shaking for 30 min until a milky white suspension was formed. Subsequently, 16 mL of 1 M NaOH solution was added with shaking until the suspension turned into a clear and colorless solution. The solution was then diluted to a final volume of 100 mL with distilled water, filtered through a 0.22 μm membrane filter, and stored at 4 °C in the dark.

Main instruments and equipment

Instrument	Model	Manufacturer
Critical point dryer	EM CPD300	Leica Microsystems, Wetzlar, Germany
Block trimmer	EM TRIM2	Leica Microsystems, Wetzlar, Germany
Ultramicrotome	EM UC7	Leica Microsystems, Wetzlar, Germany
Field-emission scanning electron microscope (FE-SEM)	SU8010	Hitachi High-Technologies, Tokyo, Japan
High-resolution field-emission transmission electron microscope (HR-TEM)	HT7700	Hitachi High-Technologies, Tokyo, Japan
Fluorescence lifetime super-resolution laser scanning confocal microscope	Stellaris 5	Leica Microsystems, Wetzlar, Germany
