# Supplementary material for: Physiological–Biochemical Characteristics and Omics Landscape of Drought Tolerance in Chlorella sp. from the Taklamakan Desert of China
Source: Int J Mol Sci. 2026 Sep 22;27(19):8439. doi: 10.3390/ijms27198439 (PMC13664879; doi:10.3390/ijms27198439)
Supplement: Supplementary file 1 [file ijms-27-08439-s001.zip › Supplementary File S3 Transcriptomic Analysis.pdf]

Detection conditions and analytical methods for transcriptomic sequencing

(1) Sample processing and preparation: *Chlorella* cell pellets from the MS treatment and the corresponding control (CK) were collected at 1 g (fresh weight) per sample, with three biological replicates. The pelleted cells were washed three times with phosphate-buffered saline (PBS) by centrifugation at 8,000 rpm for 5 min at room temperature to remove residual culture medium and surface impurities. The washed samples were immediately snap-frozen in liquid nitrogen for 15 min and subsequently stored at −80 °C until further use.

(2) RNA extraction, quality assessment, and quantification: Total RNA was extracted from each *Chlorella* sample using the TGuide S96 Plant Total RNA Extraction Kit (Tiangen, China) according to the manufacturer's instructions, and further purified with the RNA Purification Kit (Tiangen, China). The purified total RNA was assessed for concentration, purity, and RQN (RNA Quality Number) using a NanoDrop 2000 spectrophotometer (Thermo Fisher Scientific, USA) and an Agilent 5300 Bioanalyzer (Agilent Technologies, USA). RNA integrity was additionally verified by agarose gel electrophoresis. Only samples meeting the following criteria were retained for downstream analyses: total RNA yield ≥ 0.5 μg per sample, concentration ≥ 20 ng/μL, RQN > 4.5, and no visible contamination by pigments, proteins, or polysaccharides.

(3) cDNA library construction: Qualified total RNA from step (2) was used for library construction. Poly(A)$^+$ mRNA was specifically enriched from total RNA using oligo(dT)-conjugated magnetic beads via A–T base pairing. The enriched mRNA was randomly fragmented to approximately 300 bp using fragmentation buffer. First-strand cDNA was synthesized using random hexamer primers, followed by second-strand cDNA synthesis with reverse transcriptase and DNA polymerase to generate stable double-stranded cDNA. The double-stranded products were end-repaired using End Repair Mix to generate blunt ends, and an adenosine (A) tail was added to the 3′ ends for Y-shaped adapter ligation. The second-strand cDNA containing dUTP was subsequently digested with uracil-DNA glycosylase (UDG), retaining only the first-strand cDNA that preserves the original RNA strand orientation. Adapter-ligated cDNA fragments were purified and size-selected using AMPure XP beads (Beckman Coulter, USA). The selected products were PCR-amplified, and the final cDNA library was circularized using the MGIEasy kit (MGI Tech, China) to render it compatible with the DNBSEQ sequencing platform.

(4) Library sequencing: The constructed cDNA libraries were sequenced on the DNBSEQ-T7 platform (MGI Tech, China).

(5) Quality control of raw sequencing data: Raw sequencing data were first subjected to quality control, including the removal of adapter sequences and reads lacking insert fragments. Low-quality reads were filtered out if more than 50% of bases had a Phred quality score (Qphred) ≤ 20 or if the proportion of ambiguous bases (N) exceeded 10%. The remaining clean reads were further evaluated using fastp, and quality metrics including Q20, Q30, and GC content were calculated.

(6) Reference genome alignment: The quality-controlled clean reads were aligned to reference genomes and transcriptomes using HISAT2. The reference set comprised the genome and transcriptome of *Chlorella vulgaris*, the full-length transcriptome of the desert *Chlorella* strain TLD 6B, and a *de novo* assembly without reference. The resulting mapped reads were used for subsequent transcript assembly and expression quantification, and the alignment results were subjected to quality assessment.

(7) Expression quantification: Gene expression levels were quantified using RSEM, with clean reads as input. The Bowtie2 alignment module within RSEM was employed to map the clean reads, yielding raw read counts for each gene. The Expectation-Maximization (EM) algorithm was then applied to estimate expression levels for genes and transcripts, and reads

were probabilistically allocated to individual transcripts based on a statistical model. The raw counts were subsequently converted to fragments per kilobase of transcript per million mapped reads (FPKM) and normalized. A high-confidence expression matrix was retained by filtering for genes with FPKM > 0.3 in at least two time points for downstream differential expression analysis. Principal component analysis (PCA) was also performed based on the expression profiles.

(8) Differential expression analysis：Differential expression analysis between sample groups was performed using DESeq2 to identify differentially expressed genes (DEGs). The default screening criteria for significant DEGs were: false discovery rate (FDR) < 0.05 and $|\log_2 FC| \geq 1$ (corresponding to a fold change ≥ 2). Genes meeting both criteria were considered differentially expressed. Venn diagrams were generated to visualize the intersections of DEG sets between pairwise comparisons.

(9) Gene annotation and KEGG annotation of DEGs：Functional annotation of the clean reads was performed using multiple databases, including GO, KEGG, Pfam, eggNOG, NR, and Swiss-Prot. In particular, KEGG annotation was used to classify genes according to their associated pathways and functional categories.

(10) KEGG enrichment analysis of DEGs：KEGG enrichment analysis was conducted on the annotated DEGs using the SciPy package. Fisher's exact test was applied, and *p*-values were adjusted for multiple testing using the Benjamini–Hochberg (BH) procedure. Pathways with adjusted $p < 0.05$ were considered significantly enriched.

(11) Expression patterns of key DEGs and qRT-PCR validation：Heatmaps were generated to visualize the differential expression profiles of all genes within key pathways identified from the KEGG enrichment analysis. Additionally, candidate key genes from these pathways were selected for validation by quantitative real-time PCR (qRT-PCR) to confirm the RNA-seq results.

Main reagents and chemicals：

Reagent	Chemical formula	CAS No.	Specification	Source
Agarose	$C_{12}H_{18}O_9$	9012-36-6	Electrophoresis grade	Biowest, Nuaillé, France
Tris base	$C_4H_{11}NO_3$	77-86-1	≥99.0%	Aladdin, Shanghai, China
EDTA (0.5 M, pH 8.0)	$C_{10}H_{14}N_2Na_2O_8 \cdot 2H_2O$	6381-92-6	AR	Beyotime, Shanghai, China
TGuide S96 Plant Total RNA Extraction Kit	–	–	–	Tiangen, Beijing, China
RNA Purification Kit	–	–	–	Majorbio, Shanghai, China
Illumina Stranded mRNA Prep, Ligation	–	–	–	Illumina, San Diego, CA, USA
RiboCop rRNA Depletion Kit for Plants	–	–	–	Lexogen, Vienna, Austria

Preparation of Main Reagents

1.Preparation of 50× TAE stock solution:242 g of Tris base was dissolved in 800 mL of distilled water with thorough mixing. Subsequently, 100 mL of 0.5 M EDTA (pH 8.0) was added and mixed uniformly, followed by the slow addition of 57.1 mL of glacial acetic acid with continuous stirring. The solution was then brought to a final volume of 1000 mL with distilled

water.

Main instruments and equipment

Instrument	Model	Manufacturer
Tissue homogenizer	TL-48R	Wanbo, Shanghai, China
Mini centrifuge	ABSON MiFly-6	Abson, Hefei, China
High-speed refrigerated microcentrifuge	Eppendorf 5424R	Eppendorf, Hamburg, Germany
Electrophoresis apparatus	DYY-6C	Liuyi, Beijing, China
Microvolume spectrophotometer	NanoDrop 2000	Thermo Fisher Scientific, Waltham, MA, USA
Bioanalyzer	Agilent 5300	Agilent Technologies, Santa Clara, CA, USA
Fluorometer	Qubit 4.0	Thermo Fisher Scientific, Waltham, MA, USA
Thermal cycler	T100 Thermal Cycler	Bio-Rad, Hercules, CA, USA
High-throughput sequencer	DNBSEQ-T7	MGI, Shenzhen, China

Main software and tools used in this study

Software/Database	Version	Analysis	URL
Pfam database	35.0	Gene annotation	http://pfam.xfam.org/
KEGG database	2022.1	Gene and metabolite annotation	http://www.genome.jp/kegg/
eggNOG database	2020.06	Gene annotation	http://eggnogdb.embl.de/#/app/home
Swiss-Prot database	2022.1	Gene annotation	https://www.uniprot.org/
GO database	2022.0915	Gene annotation	http://www.geneontology.org/
NR database	2022.1	Gene annotation	https://www.ncbi.nlm.nih.gov/public/
GATK	3.8	SNP/Indel analysis	https://software.broadinstitute.org/gatk/download/
fastx_toolkit	0.0.14	Sequencing data quality control	http://hannonlab.cshl.edu/fastx_toolkit/
DEGSeq	1.38.0	Differential expression analysis	http://bioconductor.org/packages/stats/bioc/DEGSeq/
RSEM	1.3.3	Expression quantification	http://deweylab.biostat.wisc.edu/rsem/
GOATOOLS	0.6.5	Gene set analysis (GO enrichment)	https://github.com/tanghaibao/goatools
HISAT2	2.1.0	Sequence alignment	http://ccb.jhu.edu/software/hisat2/index.shtml
SnpEff	5.4c	Variant annotation and effect prediction	https://pcingola.github.io/SnpEff/
SciPy	1.18.0	KEGG enrichment analysis	https://github.com/scipy/scipy
Pfam database	35.0	Gene annotation	http://pfam.xfam.org/
