# Supplementary material for: Physiological–Biochemical Characteristics and Omics Landscape of Drought Tolerance in Chlorella sp. from the Taklamakan Desert of China
Source: Int J Mol Sci. 2026 Sep 22;27(19):8439. doi: 10.3390/ijms27198439 (PMC13664879; doi:10.3390/ijms27198439)
Supplement: Supplementary file 1 [file ijms-27-08439-s001.zip › Supplementary File S4 Metabolomic Analysis.pdf]

Widely targeted metabolomics analysis

(1) Sample processing and preparation:The sample processing and preparation for widely targeted metabolomics analysis were performed following the same procedures as described in Section 3.3.3 for transcriptomic sequencing analysis.

(2) Metabolite extraction:Desert *Chlorella* samples were placed in a vacuum freeze dryer and lyophilized for 12 h until completely dry. The dried samples were ground to a fine powder using a tissue homogenizer at 30 Hz for 1.5 min. Subsequently, 50 mg of the sample powder was accurately weighed and mixed with 1.2 mL of pre-cooled 70% methanol (containing internal standards) at −20°C. The mixture was thoroughly vortexed six times (30 s each, with 30-min intervals between each vortex), and then centrifuged at 12,000 rpm for 3 min. The resulting supernatant was filtered through a 0.22-μm membrane and collected as the test sample for subsequent ultra-performance liquid chromatography-tandem mass spectrometry (UPLC-MS/MS) analysis.

(3) Chromatographic and mass spectrometric conditions:Chromatographic separation was performed on an ACQUITY UPLC HSS T3 column (1.8 μm, 2.1 mm × 100 mm). The mobile phase consisted of solvent A (ultrapure water containing 0.1% formic acid) and solvent B (acetonitrile containing 0.1% formic acid). The column temperature was maintained at 40°C, with a flow rate of 0.4 mL/min and an injection volume of 4 μL. A gradient elution program was applied for sample analysis，The chromatographic and mass spectrometric conditions are shown in the table.

Gradient elution conditions of the chromatographic column for widely targeted metabolomics

Time (min)	A (%)	B (%)
0	95	5
5	35	65
6	1	99
7.5	1	99
7.6	95	5
10	95	5

Mass spectrometry conditions for widely targeted metabolomics

Mass spectrometry conditions	Positive ESI^+ parameters	Negative ESI^- parameters
Duration（min）	10	10
IonSpray Voltage（V）	5000	-4000
Temperature（°C）	550	550
Ion Source Gas1（psi）	50	50
Ion Source Gas2（psi）	60	60
Curtain Gas（psi）	35	35
Declustering Potential（V）	60	-60
MS1 Collision Energy（V）	10	-10
MS2 Collision Energy（V）	30	-30
Collision Energy Spread（V）	15	15
MS1 TOF Masses（Da）	50-1250	50-1250
MS2 TOF Masses（Da）	25-1250	25-1250

(4) Mass spectrometry data processing:The raw mass spectrometry data were first converted to mzXML format using ProteoWizard software. Peak detection, alignment, and retention time correction were then performed using the XCMS program. Peaks with a missing rate exceeding 50% were filtered out, and missing values were imputed using the K-nearest neighbors (KNN) method. Subsequently, peak areas were corrected using the support vector regression (SVR) method. After correction and filtration, the resulting peaks were identified by searching against an in-house database, integrated public databases (KEGG, HMDB, Metlin, *et al.*), and prediction databases (generated by AI algorithms), combined with the metabolic reaction network-based recursive metabolite annotation algorithm (metDNA). Following identification, metabolites with a comprehensive identification score ≥ 0.5 and a coefficient of variation (CV) < 0.3 in quality control (QC) samples were retained. Finally, positive and negative ion mode data were merged, retaining the metabolites with the highest identification confidence level and the smallest CV value, to generate the metabolite dataset for all samples (ALL-sample-data file).

(5) Sample quality assessment:The reproducibility of metabolite extraction and detection was evaluated by analyzing the total ion current (TIC) of QC samples. The stability and quality of the detection process were assessed using Pearson correlation analysis, with higher correlation coefficients (closer to 1) indicating better quality. The dispersion of the data was evaluated by the coefficient of variation (CV). A lower CV value for QC samples indicates higher experimental stability. Specifically, if the proportion of features with CV < 0.3 in QC samples exceeded 75%, the experimental data were considered to exhibit excellent stability and high sequencing quality.

(6) Principal component analysis:Principal component analysis (PCA) was performed on all samples to visualize the overall metabolic differences and variability among different treatment groups as well as within the same treatment group.

(7) Cluster analysis:Metabolite abundance data were normalized using unit variance scaling (UV) according to the formula $x' = (x - \mu) / \sigma$. Heatmaps were generated to visualize metabolite abundance, and hierarchical cluster analysis (HCA) was further performed.

(8) Differential metabolite screening:Differential metabolites were screened based on the variable importance in projection (VIP) values calculated from the OPLS-DA model, combined with *P*-values and fold change (FC). Given that the widely targeted metabolomics experiment included three biological replicates per group, a composite screening criterion was adopted to ensure biological significance: $P < 0.05$, VIP > 1.0, and FC > 2.0 (i.e., $|\log_2 FC| \geq 1$). Additionally, metabolites that were barely detectable in the control group but significantly accumulated in the treatment group were included as candidate differential metabolites if they met the criteria of FC > 5.0 and $P < 0.01$, regardless of the VIP > 1.0 threshold.

(9) KEGG annotation and enrichment analysis of metabolites:Metabolites were annotated using the KEGG database and classified according to their associated pathways and biological functions. KEGG enrichment analysis was performed using the Python SciPy package with Fisher's exact test, and *P*-values were adjusted for multiple testing. Pathways with $P < 0.05$ were considered significantly enriched.

Soluble sugar metabolomics analysis

(1) Sample preparation and processing:The preparation and processing of samples were the same as those for widely targeted metabolomics sequencing analysis.

(2) Soluble sugar extraction:A total of 100 mg of algal paste was accurately weighed using an electronic balance and transferred into a 2.0 mL centrifuge tube. Then, 700 μL of 80% (v/v) ethanol solution was added, and the mixture was extracted in a thermostatic shaker at 50 °C for 2 h. Subsequently, 700 μL of distilled water was added, and the mixture was centrifuged at

10,000 rpm for 3 min at room temperature. The supernatant was collected and transferred to a new centrifuge tube, which served as the soluble sugar sample.

(3) Chromatographic and mass spectrometric conditions:Chromatographic separation was performed on a CarboPac PA1 column (250 × 4.0 mm). The mobile phase consisted of solvent A (ultrapure water) and solvent B (100 mM sodium hydroxide solution). The column temperature was maintained at 30 °C, with an injection volume of 10 μL and a flow rate of 1.0 mL/min. The sample analysis was carried out using a gradient elution program.The gradient elution program is shown in the table below.

Mobile phase gradient conditions for carbohydrate chromatography columns

Time (min)	A (%)	B (%)
0	95	5
12	90	10
15	0	100
25	0	100
40	95	5
60	95	5

(4) Sample quality assessment and principal component analysis:Given the limited number of soluble sugar species detected, quality control was performed solely using Pearson correlation analysis and principal component analysis (PCA), with subsequent data analysis integrated into the widely targeted metabolomics dataset.

Organic acid metabolomics analysis

(1) Sample preparation:The sample preparation procedure was identical to that described for widely targeted metabolomics analysis.

(2) Organic acid extraction:A total of 100 mg of algal paste was accurately weighed using an electronic balance and transferred into a 2.0 mL centrifuge tube. Then, 1 mL of chloroform–methanol solution was added, and the mixture was extracted in a thermostatic shaker at 4 °C for 2 h, followed by static incubation on ice for 30 min. Subsequently, 600 μL of distilled water was added, and the mixture was centrifuged at 10,000 rpm for 10 min at 4 °C. The supernatant was collected into a new centrifuge tube. This extraction procedure was repeated twice, and the supernatants were combined and subjected to derivatization to obtain the organic acid samples.

(3) Chromatographic and mass spectrometric conditions:Chromatographic separation was performed on a Waters BEH C18 column (50 × 2.1 mm, 1.8 μm). The mobile phase consisted of solvent A (water containing 0.1% formic acid) and solvent B (acetonitrile containing 0.1% formic acid). The column temperature was maintained at 40 °C, with a flow rate of 0.35 mL/min and an injection volume of 2 μL. A gradient elution program was employed for sample analysis, The chromatographic and mass spectrometric conditions are summarized in the table below.

Mobile phase gradient conditions for organic acid chromatography

Time (min)	A (%)	B (%)
0	90	10
2	90	10
12	10	90
14	10	90
14.1	90	10
16	90	10

Mass spectrometric conditions for organic acid analysis

Mass spectrometry conditions	Positive ESI parameters
Sheath gas（ arb）	40
Auxiliary gas（ arb）	10
Ion spray voltage（V）	-2800
Temperature（℃）	350
Ion transfer tube temperature（℃）	320
Scan mode	Fullms-ddMS2
MS^1 scan range (m/z)	120-1500
Polarity	ESI-

(4) Sample quality assessment and principal component analysis:Given the limited number of organic acid species detected, quality control was performed solely using Pearson correlation analysis and principal component analysis (PCA), with subsequent data analysis integrated into the widely targeted metabolomics dataset.

Main reagents and chemicals

Reagent	Chemical formula	CAS No.	Specification	Source
Methanol	CH_4O	67-56-1	Chromatographic grade	Merck KGaA, Germany
Acetonitrile	C_2H_3N	75-05-8	Chromatographic grade	Merck KGaA, Germany
Formic acid	CH_2O_2	64-18-6	Chromatographic grade	Aladdin, Shanghai, China
Sodium hydroxide	NaOH	1310-73-2	AR	Sinopharm Chemical Reagent Co., Ltd., China
RNA Purification Kit	–	–	–	Majorbio, Shanghai, China

Preparation of Main Reagents

(1) Preparation of 70% methanol:Methanol (70 mL) was accurately measured and diluted to a final volume of 100 mL with distilled water.

(2) Preparation of 0.1% formic acid solution:Formic acid (1 mL) was accurately measured

and diluted to a final volume of 1000 mL with deionized water.

(3) Preparation of 0.1% formic acid in acetonitrile:Formic acid (1 mL) was accurately measured and diluted to a final volume of 1000 mL with acetonitrile.

(4) Preparation of 100 mM sodium hydroxide solution:NaOH (0.4 g) was accurately weighed, dissolved in 80 mL of distilled water with stirring until completely dissolved, and then diluted to a final volume of 100 mL with distilled water.

Main instruments and equipment

Instrument	Model	Manufacturer
Mass spectrometer	TripleTOF 6600+	SCIEX, Framingham, MA, USA
UHPLC system	LC-30A	Shimadzu, Kyoto, Japan
Centrifuge	5424R	Eppendorf, Hamburg, Germany
Thermostatic shaker	MU-G02-0448	Miulab, Taipei, China
Microbalance	MS105DM	Mettler-Toledo, Zurich, Switzerland
Centrifugal evaporator	CentriVap	LABCONCO, Kansas City, MO, USA
Vortex mixer	VORTEX-5	Kylin-Bell, Haimen, China
Ultrasonic cleaner	KQ5200E	Kunshan Ultrasonic Instruments, Kunshan, China
Pipette	Research plus	Eppendorf, Hamburg, Germany
Automated workstation	Biomek i5	Beckman Coulter, Brea, CA, USA
Plate sealer	Mini HES	Mona, Shanghai, China
Vacuum freeze dryer	Scientz-100F	Scientz Biotechnology, Ningbo, China
Ion chromatography system	ICS-5000+	Thermo Fisher Scientific, Waltham, MA, USA
UHPLC system	Vanquish	Thermo Fisher Scientific, Waltham, MA, USA
Mass spectrometer	Q Exactive	Thermo Fisher Scientific, Bremen, Germany

Main software and tools used in this study

Software/Database	Version	Analysis	URL
ProteoWizard	3.x	Raw data reading and format conversion	http://www.proteowizard.org
HMDB	5.0	Metabolite annotation	https://www.hmdb.ca/
Metlin	960K	Metabolite identification	http://metlin.scripps.edu/
